# Peptide Super-Agonist Enhances T-Cell Responses to Melanoma

**DOI:** 10.3389/fimmu.2019.00319

**Published:** 2019-03-13

**Authors:** Sarah A. E. Galloway, Garry Dolton, Meriem Attaf, Aaron Wall, Anna Fuller, Cristina Rius, Valentina Bianchi, Sarah Theaker, Angharad Lloyd, Marine E. Caillaud, Inge Marie Svane, Marco Donia, David K. Cole, Barbara Szomolay, Pierre Rizkallah, Andrew K. Sewell

**Affiliations:** ^1^T-Cell Modulation Group, Division of Infection and Immunity, Cardiff University School of Medicine, Cardiff, United Kingdom; ^2^Immunocore LTD, Oxford, United Kingdom; ^3^Department of Hematology and Oncology, Center for Cancer Immune Therapy, Herlev Hospital, University of Copenhagen, Herlev, Denmark; ^4^Systems Immunity Research Institute, Cardiff University School of Medicine, Cardiff, United Kingdom

**Keywords:** altered peptide ligand, T-cell receptor, melanoma, tumour infiltrating lymphocyte, cancer vaccine, cancer immunotherapy

## Abstract

Recent immunotherapeutic approaches using adoptive cell therapy, or checkpoint blockade, have demonstrated the powerful anti-cancer potential of CD8 cytotoxic T-lymphocytes (CTL). While these approaches have shown great promise, they are only effective in some patients with some cancers. The potential power, and relative ease, of therapeutic vaccination against tumour associated antigens (TAA) present in different cancers has been a long sought-after approach for harnessing the discriminating sensitivity of CTL to treat cancer and has seen recent renewed interest following cancer vaccination successes using unique tumour neoantigens. Unfortunately, results with TAA-targeted “universal” cancer vaccines (UCV) have been largely disappointing. Infectious disease models have demonstrated that T-cell clonotypes that recognise the same antigen should not be viewed as being equally effective. Extrapolation of this notion to UCV would suggest that the *quality* of response in terms of the T-cell receptor (TCR) clonotypes induced might be more important than the *quantity* of the response. Unfortunately, there is little opportunity to assess the effectiveness of individual T-cell clonotypes *in vivo*. Here, we identified effective, persistent T-cell clonotypes in an HLA A2^+^ patient following successful tumour infiltrating lymphocyte (TIL) therapy. One such T-cell clone was used to generate super-agonist altered peptide ligands (APLs). Further refinement produced an APL that was capable of inducing T-cells in greater magnitude, and with improved effectiveness, from the blood of all 14 healthy donors tested. Importantly, this APL also induced T-cells from melanoma patient blood that exhibited superior recognition of the patient's own tumour compared to those induced by the natural antigen sequence. These results suggest that use of APL to skew the clonotypic *quality* of T-cells induced by cancer vaccination could provide a promising avenue in the hunt for the UCV “magic bullet.”

## Introduction

Vaccination against infectious diseases has saved billions of human lives and must rank amongst the very highest achievements of mankind. The power and relative ease of therapeutic vaccination against tumour associated antigens (TAA) has been a long sought-after approach for harnessing the discriminating sensitivity of the adaptive immune system to treat cancer. The search for therapeutic cancer vaccines is now more than four decades old and has been a disappointing failure in comparison to the enormous success of prophylactic pathogen immunisation programs. Only two therapeutic cancer vaccines have been approved by the FDA and neither is widely used. The first FDA approved therapeutic cancer vaccine, a prostate cancer vaccine called Sipuleucel-t (Provenge®) (1), was controversial as although it provided minimal extensions to life, treatment failed to shrink or eliminate cancer and was ultimately unprofitable. Talimogene laherparepvec (T-VEC), a herpes simplex type 1 oncolytic viral therapy was recently approved for the treatment of advanced melanoma, is often referred to as a cancer vaccine (2). T-VEC enhances clinical responsiveness to checkpoint inhibitor therapy [reviewed in Dummer et al. (3)].

Recent successes of cancer immunotherapy have renewed interest in the enormous potential of harnessing the power of the cancer patient's own immune system to treat cancer (4). These encouraging accomplishments, revolving around the systemic blocking of T-cell checkpoints (5) or equipping patient T-cells with transgenic, chimeric cancer-targeting receptors prior to adoptive transfer (6, 7), have seen recent FDA approvals for a growing list of cancer types but with low efficacy in a subset of cancers, such as lung, gastric and colorectal malignancies. Consequently, there is an urgent need to develop new enhanced strategies to harness the immune system for cancer treatment. Successes in prophylactic vaccination against tumour antigens expressed by oncogenic viruses such as human papilloma virus (8) cannot be extended to most cancers. However, cancers where the mutational load is high, such as melanoma and lung cancers (9), are known to often present mutated non-self antigens at the tumour cell surface. These “neoantigens” can be used for successful vaccination (10, 11) but the approach is laborious and highly personalised. However, the success of vaccine strategies targeting non-self, mutated antigens provides evidence that the ultimate goal of successfully vaccinating against cancer using TAA targets that are present in all individuals across the population, could be possible. Realisation of this goal will require substantial improvements in current vaccination strategies. Attempts to enhance cancer vaccination have generally focused on three main components: antigens, adjuvants and the mode/site of delivery. The main effectors of any cancer vaccine, as for other immunotherapy successes (5–7), are probably CD8 T-cells that have the power to identify and destroy cancer cells directly. Approaches to generate such cells to date have generally focused on the magnitude of the anti-cancer CD8 T-cell response. Murine adoptive transfer experiments have demonstrated that T-cell clonotypes recognising the same antigen can exhibit widely differential efficacy *in vivo* (12). Thus, a more promising strategy for cancer vaccination might aim to enhance the *quality* of the response at the clonotypic level rather than the overall *quantity* of response. Induction of superior anti-cancer T-cell clonotypes obviously requires prior knowledge about what these clonotypes are. Unfortunately, information on the best TCR clonotypes, like information on the most effective TAA to target, is lacking. Here, we identified an effective HLA A^*^0201 (HLA A2 hereafter)-restricted clonotype in the tumour infiltrating lymphocytes (TILs) that were infused into a Stage IV melanoma patient prior to complete remission (13). This T-cell clonotype was used to generate an altered peptide ligand (APL) super-agonist that induced robust T-cell responses from the PBMC of 14/14 healthy HLA A2^+^ individuals. The T-cells induced by this APL exhibited superior anti-cancer immunity when directly compared to those induced by the natural antigen in parallel assays. Importantly, we demonstrated that T-cells induced from blood of a melanoma patient using this APL were considerably more potent at recognising autologous tumour cells than those induced by the natural peptide sequence in parallel assays. These results highlight the potential importance of considering the quality of the individual T-cell clonotypes induced during future approaches to cancer vaccination.

## Methods

### Subjects

Anonymised healthy donor blood was procured as “buffy coats” from the Welsh Blood Service (WBS) (Pontyclun, Wales, UK). TIL infusion product and peripheral blood mononuclear cells (PBMC) from metastatic melanoma patients were provided as cryopreserved samples by the Center for Cancer Immune Therapy (CCIT) (Herlev Hospital, Copenhagen, Denmark). Patient MM909.24 experienced a complete response to the TIL-based adoptive cell transfer therapy (ACT) and is cancer-free 5 years post treatment and MM1413.12 experienced a partial response after TIL-based (ACT) that is ongoing as residual disease was resected. MM909.37 succumbed to disease despite TIL therapy. Detailed information on the treatment characteristics and clinical outcomes can be found in other published studies [MM909.24 and MM909.37 in Andersen et al. (13) and MM1413.12 in Andersen et al. (14)]. Details of the patient and healthy donor samples and the assays performed in this study can be found in Table 1.

**Table 1:** Patient and healthy donor samples and the assays performed.

**Donor**	**Status**	**Assay Type**	**Figure**
1	Healthy	Candidate agonist testing ELISpot for peptide reactivity	3 3
2	Healthy	Candidate agonist testing ELISpot for peptide reactivity	3 3
3	Healthy	Candidate agonist testing Proliferation	3 4
4	Healthy	Candidate agonist testing Proliferation	3 4
5	Healthy	Candidate agonist testing Proliferation	3 4
6	Healthy	EAAGIGILTV and MTSAIGILPV line staining	4
7	Healthy	EAAGIGILTV and MTSAIGILPV line staining	4
8	Healthy	EAAGIGILTV and MTSAIGILPV line staining	4
9	Healthy	EAAGIGILTV and MTSAIGILPV line staining Clonotyping	4 S6
10	Healthy	EAAGIGILTV and MTSAIGILPV line staining Proliferation	4 4
11	Healthy	EAAGIGILTV and MTSAIGILPV line staining Killing of melanoma	4 5
12	Healthy	EAAGIGILTV and MTSAIGILPV line staining	4
13	Healthy	EAAGIGILTV and MTSAIGILPV line staining Phenotyping MTSAIGILPV tetramer staining	S4 S4 7
14	Healthy	EAAGIGILTV and MTSAIGILPV line staining MTSAIGILPV tetramer staining	5 7
MM909.37	Melanoma patient	EAAGIGILTV and MTSAIGILPV line staining Killing of autologous melanoma	8 8
MM1413.12	Melanoma patient	EAAGIGILTV and MTSAIGILPV line staining	8

### Cancer Cell Lines

T2 cells were maintained in suspension culture at 37°C in R10 media [RPMI 1640 medium supplemented with 10% foetal bovine serum (FBS), 100 U/mL penicillin, 100 μg/mL streptomycin, and 2 mM L-Glutamine (Life Technologies, Paisley, UK)]. Melanoma cell lines from patients MM909.24 and MM909.37 were cultured as adherent monolayers with R10 medium (RPMI containing 10% FBS) and detached from flasks using D-PBS with 2 mM EDTA. Melanoma line FM79 (+ HLA A2 transgene) was cultured as for MM909.24 and MM909.37.

### T-Cell Clones

**Figure 1:**
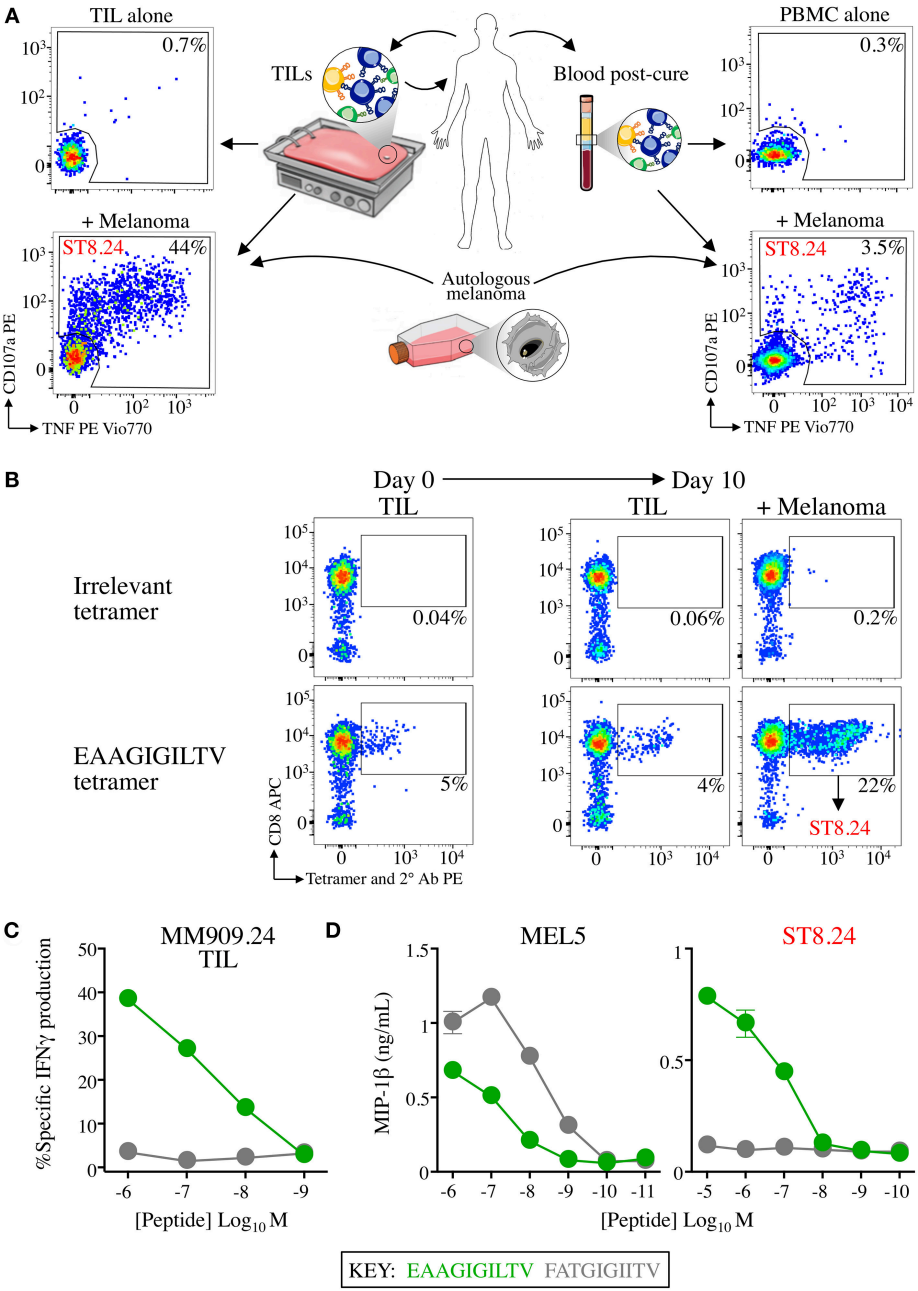
CD8 T-cell clone ST8.24 derived from patient TIL persists in patient blood after complete remission. **(A)** HLA A2-EAAGIGILTV Melan-A restricted CD8 T-cell clone ST8.24 was isolated from the TIL of a stage IV metastatic melanoma patient MM909.24 who successfully underwent tumour infiltrating lymphocyte (TIL) therapy. The TCR β chain of ST8.24 was present in the TIL infusion product, and in the blood 6 months post complete remission. **(B)** *in vitro* culture of TIL MM909.24 with autologous melanoma leads to expansion of Melan-A tetramer^+^ cells. TILs were stained prior to culture and at day 10, with irrelevant (preproinsulin, ALWGPDPAAA) and Melan-A (EAAGIGILTV) PE conjugated tetramers, using an optimised protocol (protein kinase treatment + anti-PE 1° antibody + PE conjugated 2° antibody). Percentage of cells residing in each gated population is shown. ST8.24 was amongst the expanded EAAGIGILTV tetramer^+^ T-cells. **(C)** Recognition by MM909.24 TIL of EAAGIGILTV peptide or super-agonist **F**A**T**GIGI**I**TV after 5 h using T2 cells as antigen presenting cells. The percentage of cells producing IFNγ (intracellular staining) is plotted (minus background IFNγ production by TILs alone) vs. peptide concentration. **(D)** MIP-1β ELISA of EAAGIGILTV reactive clones ST8.24 and MEL5 vs. EAAGIGILTV and **F**A**T**GIGI**I**TV peptides at the concentration range shown.

HLA A2-EAAGIGILTV (Melan-A residues/ Melanoma Antigen Recognised by T-cells (MART)-1 residues 26-35) CD8 T-cell clones from patient MM909.24 (ST8.24) or healthy donors (MEL5, MANUELA, CACTUS and EDDY) were maintained in culture in T-cell medium (R10 supplemented with 1X MEM non-essential amino acids, 1 mM sodium pyruvate, 10 mM HEPES buffer (Life Technologies), 25 ng/mL IL-15 and 200 IU/mL IL-2 (Aldesleukin, brand name Proleukin®; Prometheus, San Diego, CA). The MEL5 T-cell clone was isolated as previously described (15). Several clones expressing the same TCR as ST8.24 have been generated from patient MM909.24 on independent occasions; either from autologous melanoma reactive populations (as shown in Figure 1A); by isolation with cognate tetramer (16, 17); or by direct cloning without any prior enrichment as the TIL exhibited >20% melanoma reactivity. MANUELA, CACTUS, and EDDY were isolated based on staining with HLA A2-cognate peptide tetramers followed by cloning, then confirmation of reactivity toward exogenous peptide. Cloning was performed as previously described (18). Every 2–3 weeks, clones were expanded with 1.5 × 10^7^ irradiated (3100 cGy) allogeneic PBMCs from three donors provided by the WBS, in T-cell medium, as above but with 20 IU/mL IL-2, and as previously described (18).

### Intracellular Cytokine Staining (ICS)

TIL infusion product was co-incubated with T2 cells and a range of peptide concentrations (10^−5^-10^−12^ M) at 37°C for 5 h in R5 (RPMI containing 5% FBS) containing GolgiStop™, GolgiPlug™ (BD Bioscience, Oxford, UK) according to the manufacturer's instructions, and anti-CD107a-PE antibody (clone H483, BD Bioscience). Cells were then washed and stained with violet Live/Dead fixable dead cell stain, VIVID (Life Technologies, Paisley, UK) and for surface markers with anti-CD3 peridinin chlorophyll protein (PerCP) (clone BW264/56, Miltenyi Biotech, Bergisch Gladbach, Germany) and anti-CD8 allophycocyanin (APC)-Vio770 (clone BW135/80, Miltenyi Biotech) antibodies. Cells were prepared for ICS by incubating with Cytofix/Cytoperm™ (BD Biosciences), according to manufacturer's instructions, before staining for 20 min on ice with anti-IFNγ APC antibody (clone 45-15, Miltenyi Biotech). Cells were resuspended in FACS buffer (PBS supplemented with 2% FBS) before acquisition on BD FACS Canto II (BD Biosciences). Data was analysed using FlowJo Software (TreeStar, Ashland, OR, USA).

### Peptide Activation Assays

T-cell clones were cultured in R5 for 24 h prior to assay to reduce spontaneous activation. Assays were performed overnight in R5 in 96 U well plates with 3 × 10^5^ T-cells and 6 × 10^5^ T2 cells with and without desired peptides. The following day, supernatants were harvested, diluted with 70 μL R0 (R10 but without serum) and a half-area-well MIP-1β Enzyme-linked Immunosorbent Assay (ELISA) was carried out as per the manufacturer's instructions (R&D Systems, Minneapolis, MN). A decamer combinatorial peptide library (Pepscan Presto BV, Lelystad, The Netherlands) was stored and used as previously described (19). Individual peptides were synthesised to ≥95% purity (GL Biochem, Shanghai, China, or Peptide Protein Research Ltd, Hampshire, UK) stored at −80°C. All peptides had a free acid and free amine at the C- and N-terminus respectively. Polyclonal T-cell populations were cultured overnight in R5 in order to reduce spontaneous activation. The following day an IFNγ Enzyme-Linked ImmunoSpot (ELISpot) assay was performed, using Multiscreen plates® (Millipore, Massachusetts, MA) as per the manufacturers' instructions (Mabtech, Nacka Strand, Sweden). 5 × 10^4^ T-cells were screened against 10 candidate super-agonist peptides (described previously) at a concentration range of 10^−4^ to 10^−8^ mM. ELISpot data was collected using an ImmunoSpot® plate reader (CTL, Shaker Heights, Ohio, OH).

### Peptide T-Cell Priming

PBMCs were isolated from “buffy coats” using the SepMate protocol, as per the manufacturer's instructions (STEMCELL Technologies, Vancouver, BC, Canada). CD8 T-cells were positively purified using anti-CD8 microbeads (Miltenyi Biotech), as per the manufacturer's instructions. The CD8-negative cell population was pulsed for 1 h at 37°C with peptides of interest at a concentration of 25 μM, then irradiated (3100 cGy) to act as antigen presenting cells. CD8 T-cells were plated in 1 mL of priming media (as for T-cell media with 20 IU/mL of IL-2 and no IL-15) in 24 or 48 well plate at a density of 2 × 10^6^ or 4 × 10^6^ alongside peptide pulsed cells at a density of 3 × 10^6^ and 8 × 10^6^ per well, respectively. Anti-CD28 antibody (1 μg/mL) (clone CD28.2 Beckman Coulter) was added at the same time as plating the T-cells with the peptide pulsed PBMCs. These T-cell “primings” were fed with fresh medium tri-weekly and from day 14 onwards supplemented with 25 ng/mL of IL-15.

### Proliferation Assays

For proliferation assays, polyclonal CD8 T-cell populations isolated from HLA A2^+^ healthy individuals were labelled with 0.1 μM carboxyfluorescein succinimidyl ester (CFSE) (Thermo Fisher Scientific, Waltham, MA) prior to peptide priming. Briefly, CD8 T-cells were incubated with CFSE for 20 min at RT, and subsequently quenched with R10 medium, in order to stop the reaction. CD8 T-cells were then washed and plated in T-cell medium (T-cell media with 20 IU/mL of IL-2 and no IL-15), alongside peptide pulsed autologous irradiated CD8 negative populations, as described above.

### Peptide-MHC Tetramer Production and Staining

Soluble biotinylated peptide-MHC monomers were produced in-house as previously described (20). Tetramers were assembled and 5 × 10^4^ T-cells stained with 0.5 μg (10 μg/mL) of tetramer, with respect to pMHC component (17). An optimal tetramer staining protocol using the protein kinase inhibitor, Dasatinib (21), and primary unconjugated anti-PE antibody (22) was used as previously described (23, 24). Additionally, some samples were also stained with a secondary PE conjugated antibody to add further fluorescence to the tetramer stained cells (22). Standard staining did not use PKI or the anti-PE antibody. T-cell lines were stained with live/dead stain VIVID, anti-CD3 and anti-CD8 (APC) as above, and additionally with anti-CD4 fluorescein isothiocyanate (FITC) (clone BIT4, Miltenyi Biotech), anti-CD14 Pacific Blue (PB) (clone M5E2, BioLegend) and, anti-CD19 PB (clone HIB19, BioLegend) antibodies.

### Peptide-MHC Binding Assay

T2 cells were plated in a 96U-well plate (2 × 10^4^-1 × 10^6^ per well) in AIM-V serum-free media (Life Technologies). Peptides of interest were added at concentrations 1, 10, and 100 μM, alongside a positive control peptide that binds HLA A2 (GILGFVFTL) and two non-HLA A2 binding peptides (HLA B^*^3501-HPVGEADYFEY and HLA B^*^2705 DRASFIKNL) as negative controls. The volume of DMSO used per condition was the same and DMSO alone without peptide was used as a control. Cells were then incubated overnight at room temperature. The following day, cells were stained with anti-HLA A2 FITC antibody (clone BB7.2, BioLegend) and incubated at 37°C for 1 h. Cells were then washed in PBS and stained with VIVID, as above. Cells were then washed and resuspended in PBS ready for acquisition.

### Generation of Peptide-MHC Structures

All protein crystals were grown at 18°C by vapour diffusion via hanging drop technique. One microliter of HLA A2-**MTSA**IGIL**P**V (10 mg/mL) in crystallisation buffer (10 mM Tris pH 8.1 and 10 mM NaCl) was added to 1 μL of screen solution (20%w/v polyethylene glycol 3350, 0.2 M sodium nitrate, 0.1 M BIS-TRIS propane pH 6.5). HLA A2 crystals grown in the same conditions were crushed until no visible crystal remained using a MicroBead seed kit (Molecular Dimensions), and added to the solution. Crystallisation screens were conducted by hand, and data were collected at 100 K at the Diamond Light Source (DLS), Oxfordshire, UK at a wavelength of 0.98 using an ADSC Q315 CCD detector. Reflection intensities were estimated using XIA2 (25) and the data were analysed with SCALA and the CCP4 package (26) Structures were solved with the molecular replacement using PHASER (27). Sequences were adjusted with COOT (28) and the models refined with REFMAC5. Graphical representations were prepared with PYMOL (29). Crystal contacts were determined using PYMOL and defined as intermolecular distances <0.4. The reflection data and final model coordinates were deposited in the PDB database (HLA A2-**MTSA**IGIL**P**V, PDB: 6G3J.

### T-Cell Cytotoxic Assay

Cytotoxicity assays were performed as previously described (22). Briefly, target cells were labelled with ^51^Cr as sodium chromate (Perkin Elmer, Waltham, MA) and incubated at 37°C for 1 h then washed in R10 medium and incubated in 2 mL R10 medium for a further hour to allow excess ^51^Cr to leach from the cells. Target cells (1,000 per well) were incubated with T-cells to give desired T-cell to cancer cell ratios. Targets were incubated alone or with lysis buffer (5% Triton in H_2_O) to give spontaneous and maximum release of ^51^Cr respectively. After incubation, culture supernatant was harvested and mixed with 150 μL of Opitphase Supermix scintillation cocktail (PerkinElmer) and the release of ^51^Cr measured using a 1450 MicroBeta TriLux (PerkinElmer). For flow cytometry based killing assays, 5,000 cancer cells were co-cultured with (experimental (exp.) wells) and without (control (con.) wells) T-cells in 96 U well plates in T-cell media. Prior to harvesting, 1 × 10^5^ of 0.1 μM CFSE labelled C1R cells were added to each well to act as a reference cell population. Cells were stained with VIVID, anti-CD3 PerCP, anti-CD4 APC-Vio770 (Miltenyi Biotec), and anti-CD8 APC-Vio770 (Miltenyi Biotec) leaving viable cancer cells and CFSE labelled CIRs for analysis. Percentage killing was calculated using the following equation:

$$\begin{array}{l} \%\;Killing=100-\left(\left(\frac{exp.\;target\;cell\;events\;÷\;exp.\;CFSE\;C1R\;events}{con.\;target\;cell\;events\;÷con.\;CFSE\;C1R\;events}\right)\times 100\right) \end{array}$$

### TCR Sequencing

TCR sequencing was performed as previously described (17). RNA extraction was carried out using the RNEasy Micro kit (Qiagen, Hilden, Germany). cDNA was synthesized using the 5′/3′ SMARTer kit (Takara Bio, Paris, France). The SMARTer approach used a Murine Moloney Leukaemia Virus (MMLV) reverse transcriptase, a 3′ oligo-dT primer and a 5′ oligonucleotide to generate cDNA templates flanked by a known, universal anchor sequence at the 5′. PCR was then set up using a single primer pair consisting of TRAC or TRBC-specific reverse primer and an anchor-specific forward primer (Takara Bio, Paris, France). All samples were used for the following PCR reaction: 2.5 μL template cDNA, 0.25 μL High Fidelity Phusion Taq polymerase, 10 μL 5X Phusion buffer, 0.5 μL DMSO (all from Thermo Fisher Scientific, UK), 1 μL dNTP (50 mM each, Life Technologies, Paisley, UK), 1 μL of each primer (10 μM), and nuclease-free water for a final reaction volume of 50 μL. Subsequently, 2.5 μL of the first PCR products were taken out to set up a nested PCR as above, using a nested primer pair. All nested primers were flanked with illumina indexes. For both PCR reactions, cycling conditions were as follows: 5 min at 94°C, 30 cycles of 30 s at 94°C, 30 s at 63°C, 90 s at 72°C, and a final 10 min at 72°C. The final PCR products were loaded on a 1% agarose gel and purified with the QIAEX II gel extraction kit (Qiagen, Hilden, Germany). Purified products were sequenced on an Illumina MiSeq instrument using the MiSeq v2 reagent kit (Illumina, Cambridge, UK).

## Results

### Generation of Optimal Agonists for a T-Cell Clone Generated From a Patient That Underwent Successful TIL Therapy

Analysis of T-cells that responded to autologous tumour within the TIL infusion product used to cure an HLA A2^+^ stage IV melanoma patient (code MM909.24), and also in the blood 6 months post cure, identified several persistent TCR clonotypes (16, 17). Single cell cloning from the TIL infusion product produced a monoclonal population of one of these persistent T-cells, ST8.24 (Figure 1A demonstrates the approach). ST8.24 was found to recognise the HLA A2-restricted, Melan-A-derived peptide EAAGIGILTV, a common tumour associated antigen expressed by the majority of melanoma tumours (30). Co-incubation of TIL infusion product with the autologous tumour for 10 days demonstrated that a significant portion (22%) of the tumour-expanded TIL infusion product exhibited reactivity to EAAGIGILTV peptide (Figure 1B); ST8.24 was included in this EAAGIGILTV tetramer-positive population. Our previous work on T-cells that recognise EAAGIGILTV identified a novel super-agonist peptide, **F**A**T**GIGI**I**TV (bold underlined amino acids are not found in the germline-encoded natural TAA protein sequence) that acted as a super-agonist of melanoma-reactive T-cell clone MEL5 that was grown from a healthy donor (31). The **F**A**T**GIGI**I**TV peptide induced greater CD8 T-cell responses to the EAAGIGILTV natural sequence in 6/10 healthy donors (31). **F**A**T**GIGI**I**TV-primed T-cells also exhibited enhanced tumoricidal activity when compared to EAAGIGILTV-primed T-cells (15, 31). We tested how the **F**A**T**GIGI**I**TV peptide activated the TIL infusion product used to successfully cure patient MM909.24. The **F**A**T**GIGI**I**TV peptide failed to induce IFNγ production from this TIL infusion product (Figure 1C). While results confirmed that **F**A**T**GIGI**I**TV was a more potent agonist of the MEL5 T-cell clone, this peptide failed to activate the ST8.24 T-cell clone that was present in the TIL infusion product and persisted in patient blood after complete remission (Figure 1D). The promising results observed in the majority of donors with the **F**A**T**GIGI**I**TV super-agonist peptide designed using the healthy donor-derived MEL5 T-cell encouraged us to search for an optimal agonist peptide for the ST8.24 T-cell clone by PS-CPL screening (32, 33).

### Super-Agonist Peptide Can Induce Larger Populations of EAAGIGILTV-Specific T-Cells From Donor PBMC Than the Natural Antigen Leading to Enhanced Killing of Melanoma

**Figure 2:**
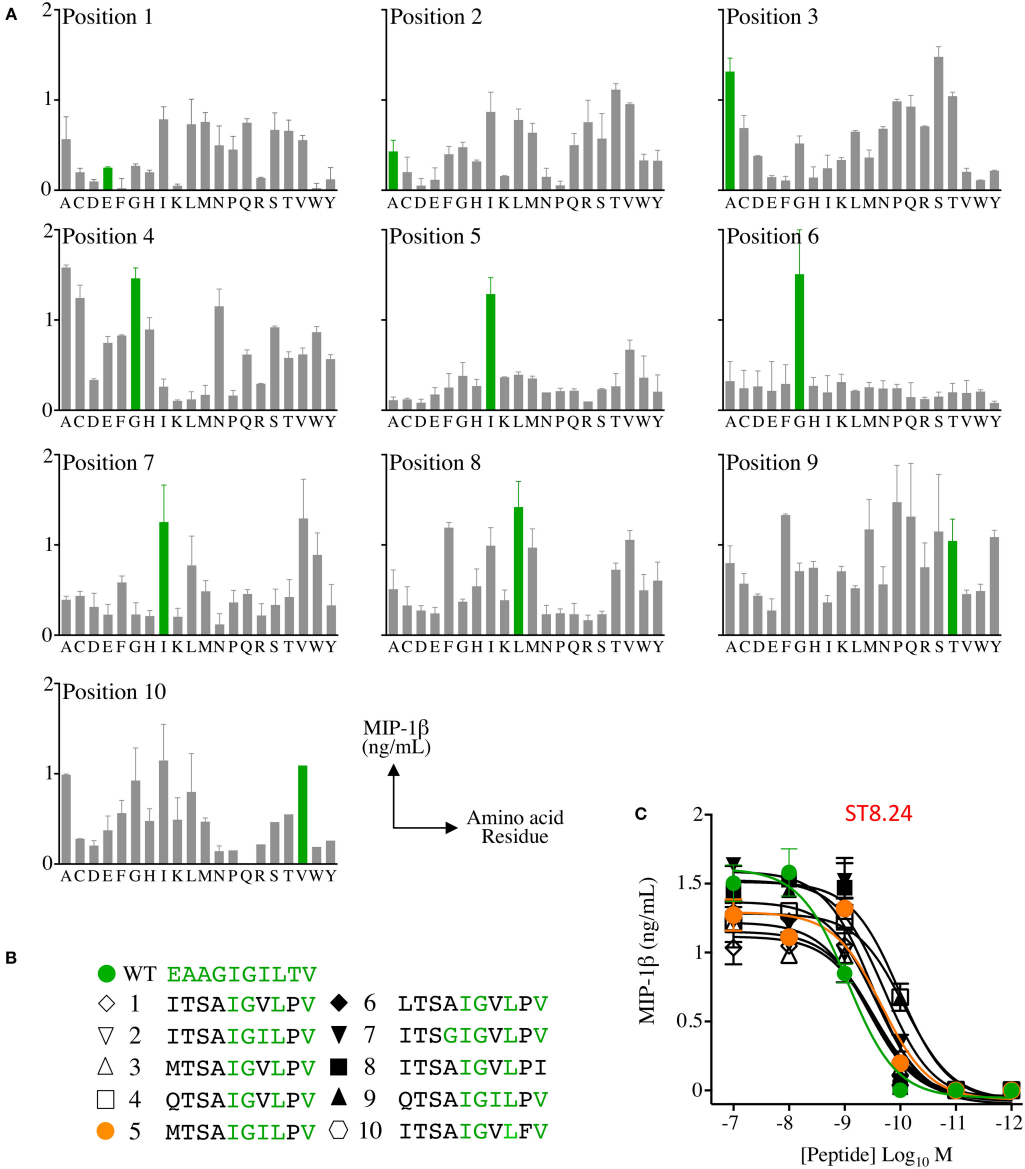
Combinatorial peptide library (CPL) screening of T-cell clone ST8.24 allowed candidate super-agonist peptides to be designed. **(A)** Decamer CPL screen with melanoma and EAAGIGILTV peptide reactive CD8 clone ST8.24, showing the preferred amino acid residue landscape (EAAGIGILTV residues shown in green). **(B)** Data from the CPL was used to generate a list of potential super-agonists. Candidate super-agonist peptides are ranked from 1 to 10 with number 1 predicted to be the most likely to be recognised by ST8.24. Amino acids residues shared with the Melan-A peptide are shown in green. **(C)** Peptide titrations of 10 candidate super-agonist and EAAGIGILTV peptides using clone ST8.24. Results expressed as MIP-1β in ng/mL, minus the MIP-1β value of T-cells incubated alone. Non-linear curves of best-fit are shown.

**Figure 3:**
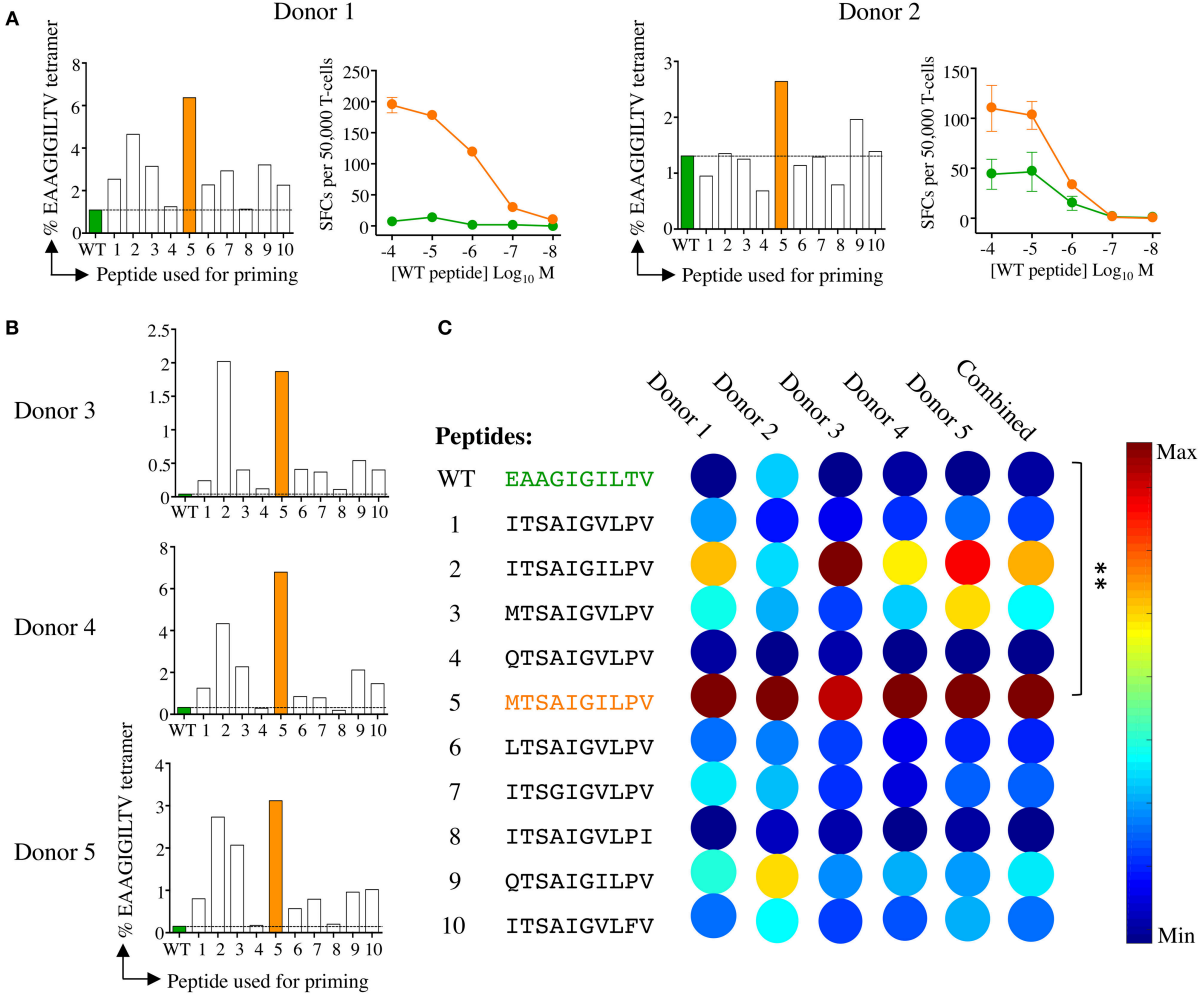
Melan-A tetramer staining of CD8 T-cells primed from healthy HLA A2^+^ donors with candidate super-agonist peptides. **(A)** CD8 T-cells isolated from five healthy donors were primed with wild-type (WT) EAAGIGILTV Melan-A peptide, or one of 10 candidate super-agonists (sequences shown in C), and cultured for 2 weeks. Primed T-cells were stained with EAAGIGILTV PE-conjugated tetramers, using an optimised protocol (protein kinase inhibitor treatment + tetramer + anti-PE 1° antibody). Percentage of EAAGIGILTV-tetramer positive cells is shown in bar charts. The dashed line aligns with the percentage of tetramer^+^ cells for the EAAGIGILTV peptide primed cells. Line graphs show reactivity to WT peptide EAAGIGILTV (at the range of concentrations indicated) of CD8 T-cells primed with EAAGIGILTV and **MTSA**IGIL**P**V from donors 1 and 2 in an IFNγ ELISpot. Results are shown as number of spot forming units (SFU) per 50,000 T-cells. **(B)** Percentage of EAAGIGILTV-tetramer positive cells primed in three further donors (as in **A**). **(C)** Heat bars were generated using MATLAB R2017b and based on the percentage of EAAGIGILTV-tetramer positive cells (A&B), with a heat bar for all donors combined shown on the right. The colour indicates the relative magnitude of priming, according to the key. Priming with peptides **MTSA**IGIL**P**V generated significantly more EAAGIGILTV tetramer positive cells when compared to priming with EAAGIGILTV (**p < 0.01). Associated flow cytometry data are shown in Supplementary Figure 1.

Raw data from the ST8.24 PS-CPL screen were used to generate a ranked list of the top 10 potential ST8.24 agonists predicted to activate ST8.24 (Figure 2). These peptides were selected using a CPL-based peptide searching algorithm (available at https://picpl.arcca.cf.ac.uk/loginform.php) applied to the decamer peptide universe, and thus they represent the best possible *in silico* predicted peptides for a given clone (34). CD8 T-cell clone ST8.24 showed good sensitivity to the 10 candidate super-agonist peptides when tested by peptide titration (concentration range 10^−7^-10^−12^ M) by MIP-1β ELISA (Figure 2C). These 10 candidate super-agonists were taken forward for further study on how APL might affect the priming of EAAGIGILTV-specific T-cells from two HLA A2^+^ healthy donors. For these experiments, CD8 T-cells from each donor were co-cultured with candidate super-agonist peptides for 14 days as described in the methods section above and tested for reactivity using HLA A2-EAAGIGILTV tetramers. In both donors, peptide 5 (**MTSA**IGIL**P**V) induced the largest population of HLA A2-EAAGIGILTV tetramer positive cells compared to the other 9 candidate super-agonists and the natural EAAGIGILTV itself (Figure 3A, Supplementary Figure 1). Functional comparison in IFNγ ELISpot showed that the cells primed with **MTSA**IGIL**P**V were capable of recognising the EAAGIGILTV natural peptide. Encouraged by these initial results, we examined priming by all 10 APL and the wild-type peptide in three further donors. As before, the **MTSA**IGIL**P**V was most potent at priming EAAGIGILTV-tetramer^+^ T-cells in donors 4 and 5 (Figure 3B). In the remaining donor (number 3) **MTSA**IGIL**P**V came a close second behind the **ISTA**IGIL**P**V sequence. The percentage of EAAGIGILTV-tetramer^+^ cells primed across these 5 individuals were used to generate a heat bar for the 10 peptides (Figure 3C). The clear superiority of the **MTSA**IGIL**P**V caused us to focus on this peptide going forward.

**Figure 4:**
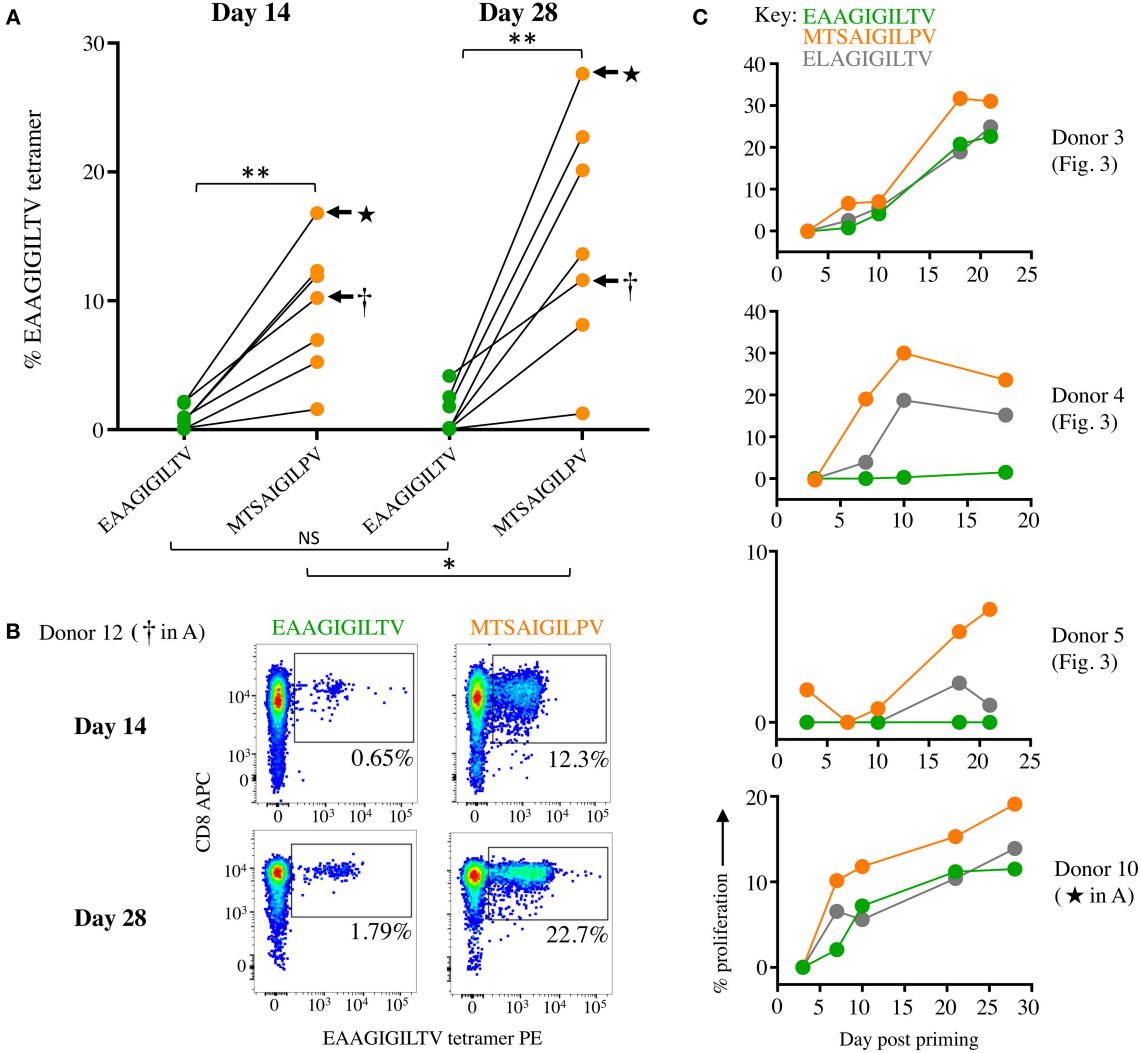
Priming CD8 T-cells from healthy donors with super-agonist peptide **MTSA**IGIL**P**V elicits significantly more EAAGIGILTV-tetramer positive cells. **(A)** CD8 T-cells from seven additional healthy donors (6-12) were primed with wild type EAAGIGILTV Melan-A peptide or super-agonist **MTSA**IGIL**P**V. The magnitude of reactive cells was tested at day 14 and 28 by EAAGIGILTV-tetramer staining, with percentage tetramer^+^ cells expressed graphically (associated flow cytometry plots shown in Supplementary Figures 2, 3). The difference in magnitude of EAAGIGILTV-tetramer positive cells elicited by the super-agonist peptide was significantly greater than EAAGIGILTV primed cells at both time points (paired one-tailed *t*-test). (NS ≥0.05 *p ≤ 0.05 **p ≤ 0.01). **(B)** Flow cytometry plots for one donor from **(A)** with percentage EAAGIGILTV-tetramer^+^ cells shown. **(C)** CFSE proliferation of T-cells primed with wild-type EAAGIGILTV, heteroclitic E**L**AGIGILTV, or super-agonist peptide **MTSA**IGIL**P**V. T-cells were sampled at multiple time points and background proliferation (CFSE^low^) in the no peptide (DMSO) condition subtracted from the CFSE^low^ cells in the peptide conditions to give percentage proliferation.

We next compared the priming of EAAGIGILTV-specific T-cells in a further 7 different HLA A2^+^ donors with the **MTSA**IGIL**P**V APL and the natural sequence over 14 and 28 days (Figure 4A). **MTSA**IGIL**P**V elicited the expansion of significantly more EAAGIGILTV-tetramer specific cells after 14 days than the EAAGIGILTV peptide itself (Paired one tailed *T*-test, *P* ≤ 0.01 ^**^) in all donors (Figure 4A, Supplementary Figure 2). Furthermore, after 28 days it was noted that the size of HLA A2-EAAGIGILTV tetramer positive cell population increased significantly in the **MTSA**IGIL**P**V primed population in all seven donors (*P* ≤ 0.05 ^*^) (Figure 4A, Supplementary Figure 3). Figure 4B shows a representative staining. The superior cell expansion with the **MTSA**IGIL**P**V peptide prompted us to set up comparative CFSE proliferation assays alongside the natural EAAGIGILTV peptide and the widely used heteroclitic analogue peptide E**L**AGIGILTV that was designed to enhance HLA binding (30) and that has been used in clinical trials with little success (35). Cells primed with **MTSA**IGIL**P**V proliferated to a greater degree compared to the EAAGIGILTV and E**L**AGIGILTV peptides in all donors tested (Figure 4C). Phenotypic analysis of **MTSA**IGIL**P**V (4.4% tetramer^+^) and EAAGIGILTV (0.6% tetramer^+^) peptide primed T-cells from donor 13 showed that the enhanced expansion of EAAGIGILTV-specific T-cells seen with the **MTSA**IGIL**P**V peptide at day 28 was not associated with accelerated exhaustion based on expression of CD45RA, CD45R0, CCR7, and CD27, as the EAAGIGILTV primed T-cells exhibited a very similar phenotype (Supplementary Figure 4). Overall, we conclude that the **MTSA**IGIL**P**V peptide expands substantially larger populations of EAAGIGILTV-specific T-cells than the EAAGIGILTV peptide itself in 13/13 donors tested and that these T-cell populations exhibited improved recognition of the natural epitope.

**Figure 5:**
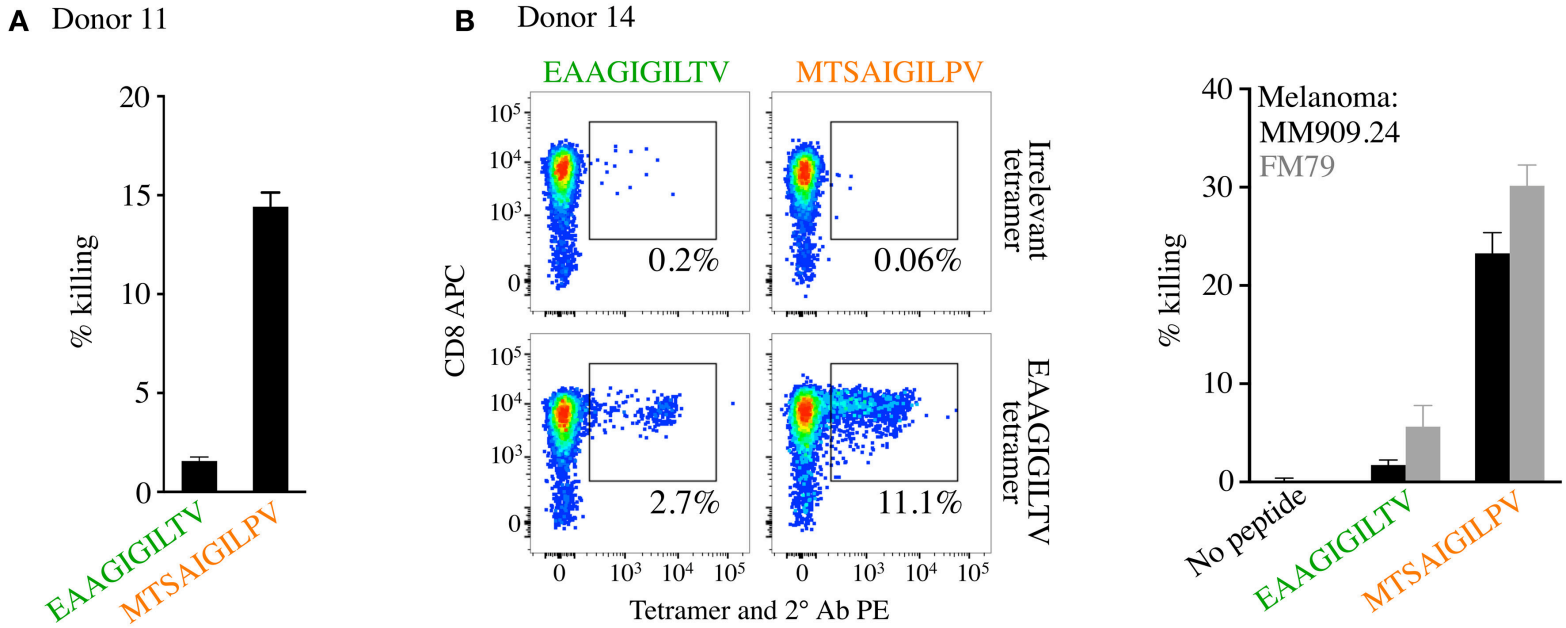
CD8 T-cells isolated from healthy HLA A2^+^ donors primed with super-agonist **MTSA**IGIL**P**V exhibit superior melanoma killing. CD8 T-cells from both donors 11 and 14 were primed with **MTSA**IGIL**P**V and EAAGIGILTV peptides and the magnitude of EAAGIGILTV-tetramer positive cells determined on day 14 and 28 for donor 11 (Figure 4, and associated flow cytometry plots in Supplementary Figures 2, 3) and on day 28 only for donor 14 (this figure). **(A)** Chromium release cytotoxicity assay performed for the T-cell lines generated from donor 11, with melanoma from patient MM909.24. **(B)** Peptide priming data (left panel) and flow cytometry based killing of melanoma tumours FM79 (+HLA A2 transgene) and MM909.24 with the CD8 T-cell lines from donor 14. A ratio of 1 T-cell to 1 tumour cell was used for both assays.

We next performed killing assays to assess whether the enhanced expansion of EAAGIGILTV specific T-cells seen with the **MTSA**IGIL**P**V peptide translated to improved cytotoxicity of melanoma cells. T-cell lines from donor 11 from the original 12 donors (Figure 4A, Supplementary Figures 2, 3), and an additional donor (number 14, **MTSA**IGIL**P**V 11.1% tetramer^+^ compared to 2.7% for EAAGIGILTV, Figure 5), were used to target allogeneic HLA A2^+^ melanomas. The **MTSA**IGIL**P**V primed lines from donors 11 and 14 killed significantly more cancer cells than the EAAGIGILTV primed lines (Figure 5). In light of this, we next sought to examine why **MTSA**IGIL**P**V-primed T-cells might expand to greater numbers.

### The Super-Agonist Peptide Exhibits Improved Binding to HLA, and Is a Structural Mimic of EAAGIGILTV

**Figure 6:**
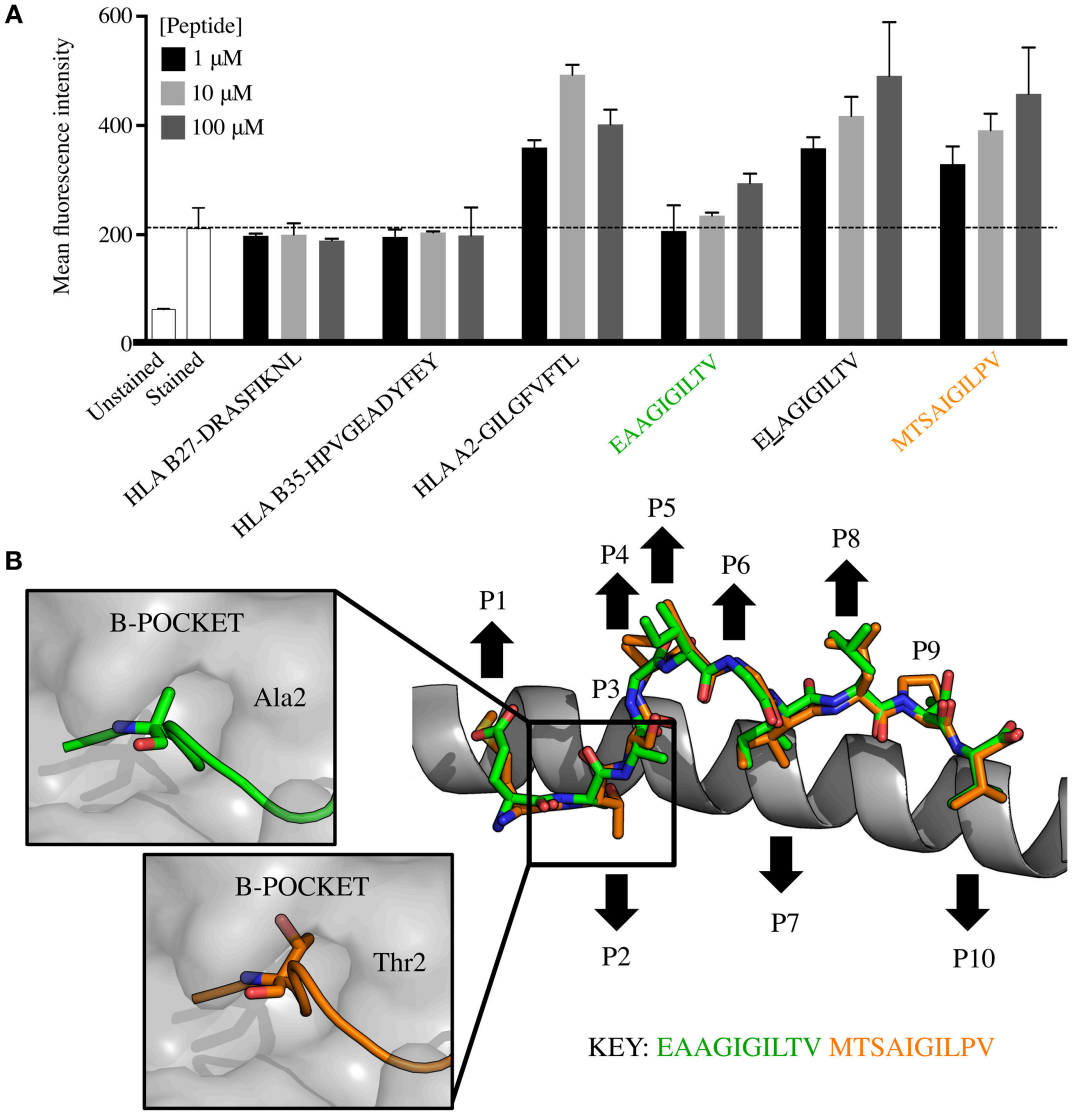
HLA A2-**MTSA**IGIL**P**V is a structural mimic of HLA A2-EAAGIGILTV that exhibits improved binding to HLA. **(A)** T2 cells (TAP deficient HLA A2^+^) were incubated overnight in serum-free media with EAAGIGILTV and **MTSA**IGIL**P**V at 1, 10, and 100 μM, alongside a panel of control peptides including HLA A2 binding peptides E**L**AGIGILTV (heteroclitic L at positon 2), GILGFVFTL (from M1 of influenza), HLA B*2705 binding peptide DRASFIKNL (from collagen) and HLA B*3501 HPVGEADYFEY (from EBV). Experiment was carried out in duplicate and results are expressed as the mean fluorescence intensity (MFI) of HLA A2 expression. The dashed line shows the MFI staining in the absence of exogenously supplied peptide. Associated flow cytometry plots are displayed in Supplementary Figure 5. **(B)** Structural analysis of the HLA A2-**MTSA**IGIL**P**V complex compared to HLA A2-EAAGIGILTV. The HLA α1 helix is shown as grey cartoon with the **MTSA**IGIL**P**V (orange sticks) and EAAGIGILTV (green sticks) superimposed. Black arrows demonstrate the upward facing solvent exposed and downward facing buried residues in each peptide. Boxes to the left show the interaction between the HLA A2 B-pocket (grey surface) and EAAGIGILTV (green cartoon and sticks) and **MTSA**IGIL**P**V (orange cartoon and sticks).

One possible explanation for why the **MTSA**IGIL**P**V peptide induced more EAAGIGILTV-specific T-cells than the EAAGIGILTV peptide itself might be because it exhibits enhanced binding to the HLA A2 restriction element and would therefore be present on the cell surface at higher copy number when supplied exogenously. We compared HLA binding of both peptides to that of the E**L**AGIGILTV sequence that was specifically designed to improve this property by addition of the optimal anchor at position (p)2 (30). **MTSA**IGIL**P**V exhibited substantially improved binding compared to EAAGIGILTV, binding almost as well as the E**L**AGIGILTV sequence (Figure 6A). The improved binding of **MTSA**IGIL**P**V and E**L**AGIGILTV to HLA A2 likely means that these peptides will be present at higher surface density than the natural peptide in comparative assays. To further explore the nature of HLA A2-**MTSA**IGIL**P**V interaction and peptide presentation mode, we solved the structure of the binary pHLA complex at 2.45 Å (Table 2, Figure 6B). The optimal position 2 anchors for HLA A2 are L, M and I so it was not clear why the **MTSA**IGIL**P**V exhibited such strong HLA binding. However, the structural analysis demonstrated that, although the total number of contacts between the peptide and HLA-binding groove were only marginally greater for **MTSA**IGIL**P**V compared to EAAGIGILTV (136 and 134 respectively), the longer side chain of threonine at position 2 in **MTSA**IGIL**P**V protruded deeper into the HLA B-pocket than alanine at position 2 in EAAGIGILTV (Figure 6B). This enhanced tethering at the N-terminal anchor position likely plays a chief role in enabling **MTSA**IGIL**P**V to form a more stable interaction with HLA A2, contributing to the greater observed immunogenicity of this peptide compared to EAAGIGILTV. Despite differing in amino acid sequence at 5/10 positions, the structural analysis also demonstrated that **MTSA**IGIL**P**V is a surprisingly close structural mimic of EAAGIGILTV (root mean square deviation = 0.38), sharing a virtually identical peptide backbone conformation. The peptides were also remarkably similar in terms of buried and exposed peptide side chains, with peptide residues 4, 5, and 6 forming the outward facing central bulge in the peptide, and residues 1 and 8 also pointing away from the HLA surface as additional potential TCR contact sites. Thus, the structural analysis provides a molecular basis for why **MTSA**IGIL**P**V is more stably presented than EAAGIGILTV, and why T-cells primed with **MTSA**IGIL**P**V can recognise the tumour associated EAAGIGILTV peptide. This structural analysis provides a molecular basis for why **MTSA**IGIL**P**V is more stably presented than EAAGIGILTV, and why T-cells primed with **MTSA**IGIL**P**V can recognise the tumour associated EAAGIGILTV peptide.

**Table 2:** Data collection and refinement statistics.

**PDB code**	**6G3J**
**DATA COLLECTION**	
DLS Beamline	I24
Space group	C 1 2 1
Wavelength (Å)	0.96859
**CELL DIMENSIONS**	
*a, b, c* (Å)	202.94, 50.28, 119.07
α, β, γ (°)	90.0, 122.86, 90.0
Resolution (Å)	2.45–41.05
Outer Shell (Å)	2.51–2.45
*R*_merge_ (%)	10.4 (115.5)
*R_*meas*_* (%)	12.3 (135.4)
CC1/2	0.993 (0.594)
*I* / *σI*	9.1 (1.0)
Completeness (%)	98.7 (99.3)
Multiplicity	3.5 (3.6)
Unique reflections	37,034 (2,764)
Wilson B Factor (Å^2^)	61.8
**REFINEMENT**	
R-work reflections	35,216
R-free reflections	1,818
R_work_/R_free_	22.3/27.0
**R.M.S. DEVIATIONS**	
Bond lengths (Å)	0.018
Bond Angles (°)	1.9
Coordinate Error	0.331
Mean B value (Å^2^)	84.8
**RAMACHANDRAN STATISTICS**	
Favoured/Allowed/Outliers	725/27/5
(%)	95.8/3.6 0.7/

*One crystal was used for determining each structure*.

*Figures in brackets refer to outer resolution shell*.

*Coordinate Estimated Standard Uncertainty error calculated based on maximum likelihood statistics*.

### Super-Agonist Tetramer Stains EAAGIGILTV Specific T-Cells With Greater Intensity

**Figure 7:**
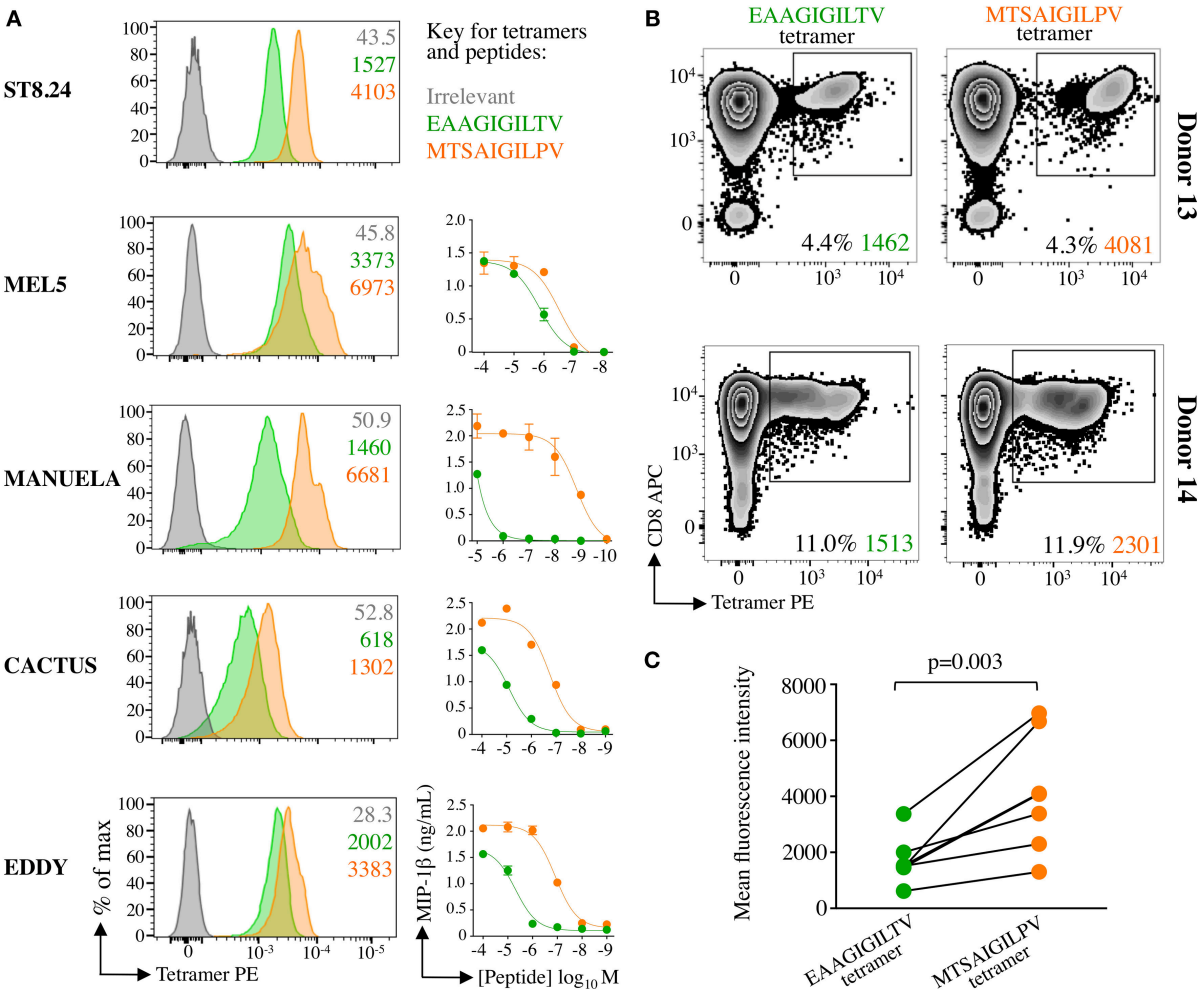
Super-agonist tetramer binds EAAGIGILTV specific T-cells with greater fluorescent intensity. **(A)** pMHC-tetramer staining of five EAAGIGILTV clones derived from healthy donors (MEL5, CACTUS, EDDY and MANUELA) or a metastatic melanoma patient (ST8.24). The mean fluorescence intensity of staining (MFI) is displayed for irrelevant (ALWGPDPAAA from preproinsulin), EAAGIGILTV and **MTSA**IGIL**P**V tetramers. Tetramer staining was carried out using a standard protocol (MEL5, CACTUS, and EDDY), with PKI (ST8.24) or with PKI + anti-PE 1° antibody + PE conjugated 2° antibody (MANUELA) in order to give clear EAAGIGILTV tetramer staining (22). Line graphs indicate sensitivity to EAAGIGILTV peptide or **MTSA**IGIL**P**V super-agonist at the concentrations indicated in an overnight activation assay followed by MIP-1β ELISA. **(B)** pMHC tetramer staining of EAAGIGILTV or **MTSA**IGIL**P**V peptide primed polyclonal CD8 T-cell populations isolated from healthy donors 13 and 14. T-cells were stained using an optimised protocol: PKI + PE tetramer + 1° + 2° antibodies. The percentage of gated cells and their MFI is displayed. **(C)** Accumulated mean florescent intensity (MFI) of all EAAGIGILTV-CD8 T-cell clones and polyclonal CD8 T-cell populations used. The difference in MFI between EAAGIGILTV-tetramer and **MTSA**IGIL**P**V-tetramer staining was statistically significant (*P* = 0.003, paired one-tailed *T*-test).

We have previously established that staining with peptide-MHC multimers is dependent on the TCR-pMHC affinity (17, 22, 36) and that optimised staining procedures are required for staining functional T-cells with very weak TCRs such as those that predominate in autoimmune T-cell populations (24). Consequently, the intensity of staining with pMHC multimers can provide an indication of TCR affinity at the population level. We next examined how HLA A2-**MTSA**IGIL**P**V and HLA A2-EAAGIGILTV tetramers stained EAAGIGILTV-specific T-cell clones and polyclonal T-cell populations. T-cell clones ST8.24, MEL5, MANUELA, CACTUS, and EDDY exhibited greater fluorescence intensity with **MTSA**IGIL**P**V tetramers relative to the tetramers bearing the natural EAAGIGILTV sequence (Figure 7A). All five of the aforementioned T-cell clones recognised the **MTSA**IGIL**P**V peptide more sensitively than the EAAGIGILTV peptide in peptide titration assays (ST8.24: Figure 2, others: Figure 7A). Additionally, T-cell lines from donors 13 and 14 also stained with the super-agonist tetramer with improved intensity relative to the wild-type EAAGIGILTV tetramer (Figure 7B). Taken together the improved staining seen with the **MTSA**IGIL**P**V tetramer was statistically significant (*p* = 0.003, Figure 7C) and as equivalent concentrations of HLA A2-**MTSA**IGIL**P**V and HLA A2-EAAGIGILTV tetramers were used for staining the T-cells, these results suggest that the associated TCRs exhibit stronger interactions with the super-agonist peptide. These data combined with the enhanced HLA binding of **MTSA**IGIL**P**V peptide, provide insight into the mechanism underlying the improved priming of EAAGIGILTV-specific T-cells by the super-agonist peptide across all donors tested.

### Super-Agonist Peptide Prime Melanoma Patient T-Cells With Improved Efficacy

**Figure 8:**
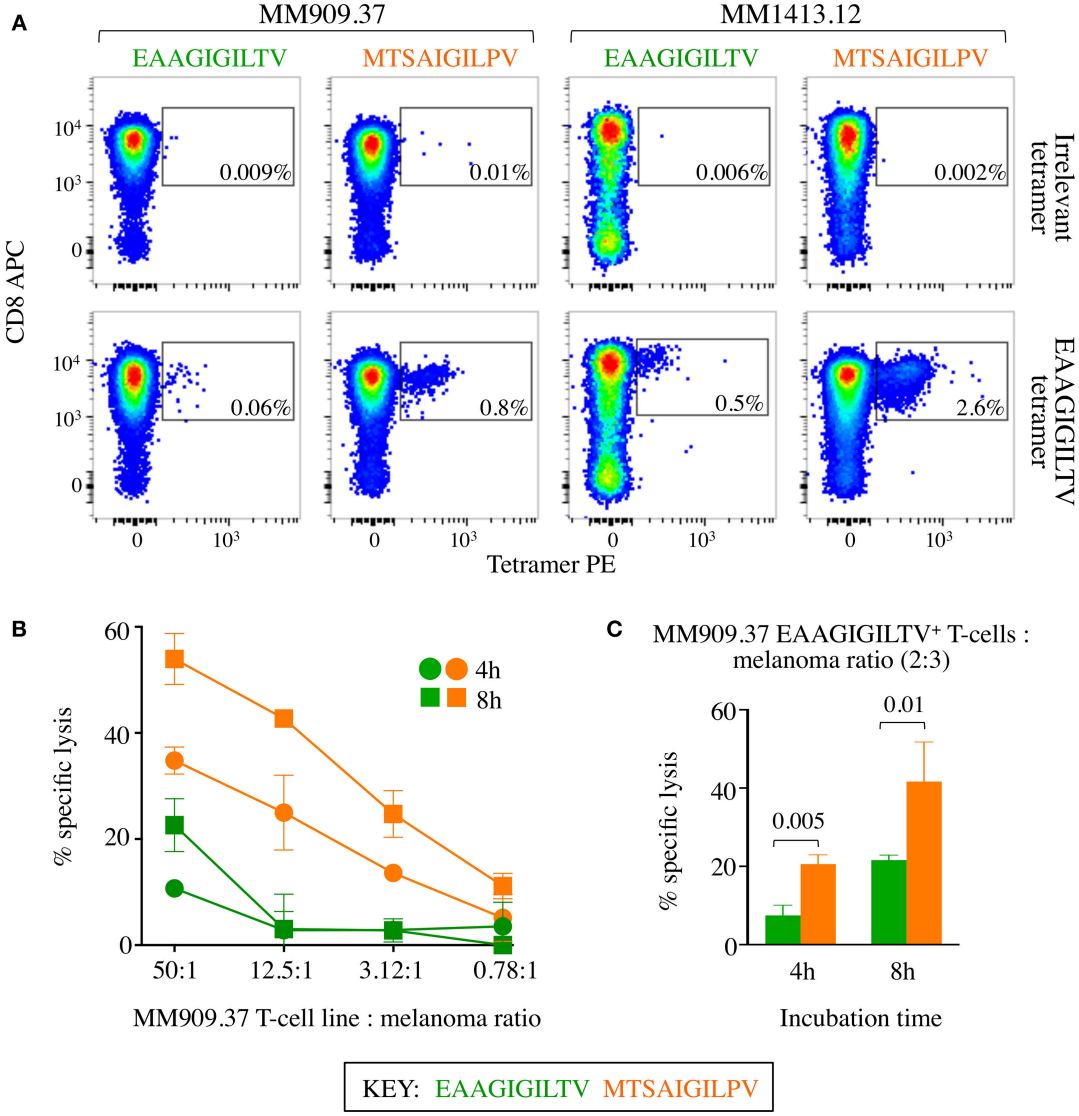
CD8 T-cells from melanoma patients primed with super-agonist **MTSA**IGIL**P**V resulted in superior autologous melanoma killing. **(A)** 4 × 10^5^ CD8 T-cells from metastatic melanoma patients MM909.37 and MM1413.12 were primed with WT EAAGIGILTV or super agonist **MTSA**IGIL**P**V peptides. MM909.37 is now deceased having not responded to therapy. EAAGIGILTV and irrelevant (ALWGPDPAAA from preproinsulin) tetramer staining was performed at day 28, with percentage of tetramer^+^ cells displayed. **(B)** Chromium release cytotoxicity assay performed for the T-cell lines from MM909.37 using autologous melanoma. The T-cell line to melanoma cell ratio displayed is based on total T-cell number. Insufficient cells were available from patient MM1413.12 to perform the killing assay. **(C)** Cytotoxicity assay as in **(B)**, but with cell numbers adjusted according to EAAGIGILTV tetramer positivity shown in **(A)**, to give two EAAGIGILTV tetramer^+^ cell per three autologous melanoma cells, for both the EAAGIGILTV and **MTSA**IGIL**P**V primed T-cell lines (thus a different number of total cells was used from each primed line). *P*-values are displayed for an unpaired one-tailed *t*-test.

The **MTSA**IGIL**P**V super-agonist peptide was capable of inducing larger EAAGIGILTV-specific T-cell responses in 14/14 healthy donors and the resulting T-cell lines from two donors that were tested exhibited improved killing of melanoma. We reasoned that a real test for this peptide would be to examine whether **MTSA**IGIL**P**V was better at inducing melanoma specific T-cells from the blood of cancer patients. To examine this aspect, we used samples from two HLA A2^+^ metastatic melanoma patients that were enrolled in clinical trials at the CCIT at Herlev hospital Copenhagen. Patient MM909.37 succumbed to disease following TIL-based adoptive cell therapy (13). Patient MM1413.12 experienced a partial response to TIL therapy and subsequently the remainder of their tumour resected, the patient is currently tumour-free with no sign of recurrence. CD8 T-cells isolated from PBMC samples donated before TIL treatment were primed with **MTSA**IGIL**P**V or EAAGIGILTV peptides. Priming of patient-derived T-cells with **MTSA**IGIL**P**V super-agonist peptide elicited a greater magnitude of EAAGIGILTV-tetramer positive cells than priming with the wild type peptide EAAGIGILTV in both donors, 0.8% compared to 0.06% in patient MM909.37 and 2.6% compared to 0.5% in patient MM1413.12 (Figure 8A). For patient MM909.37 there was enough sample to examine how the primed T-cells responded to the patient's own tumour line. T-cells primed with **MTSA**IGIL**P**V peptide demonstrated superior lysis of the autologous tumour line compared to T-cells primed with EAAGIGILTV at a range of T-cell to tumour ratios during a chromium release assay (Figure 8B). Moreover, on a per cell basis (two EAAGIGILTV-tetramer positive cells per three melanoma tumour cells) **MTSA**IGIL**P**V-primed cells were significantly better at killing autologous tumour after 4 and 8 h of incubation (*P* ≤ 0.005 and *P* ≤ 0.01) compared to EAAGIGILTV-primed cells (Figure 8C). Unfortunately, PBMCs from melanoma patients prior to treatment were at a premium and we did not generate sufficient EAAGIGILTV-specific cells from patients MM909.37 and MM1413.12 for clonotyping. Analyses of EAAGIGILTV specific TCRs from EAAGIGILTV and **MTSA**IGIL**P**V primed T-cells from donor 9, who had large EAAGIGILTV-specific populations (Figure 4), revealed each peptide induced T-cells with different TCR clonotypes. More than half the TCRs from both lines expressed the TRAV12-2 gene in TCR α chain, which is a bias associated with HLA A2-EAAGIGILTV specific T-cells (37, 38), but only 2 CDR3α and 1 CDR3β of the total 162 unique CDR3s were shared between the EAAGIGILTV and **MTSA**IGIL**P**V lines (Supplementary Figure 6). Overall, we conclude that super agonist peptide **MTSA**IGIL**P**V can prime a greater quantity of T-cells than is generated by the natural sequence EAAGIGILTV and that these T-cells can have different clonotypes that may be of a higher *quality* than those primed with the natural antigen.

## Discussion

Several hurdles need to be overcome before general, non-personalised cancer vaccines become a reality. First, the best ubiquitous targets that are widely [over]expressed in cancers but not healthy cells and that are presented at the cell surface in HLA class I molecules must be identified. Second, strategies must be developed to successfully break self-tolerance to such antigens. Central tolerance is known to delete T-cells that bear a TCR with high affinity for self-antigens in order to protect against autoimmunity (39). This process removes the very best TCRs for recognising TAA leaving those that are on average have a ~5-fold weaker affinity than receptors that recognise pathogen-derived, non-self-antigens (40, 41). TCR affinity is known to govern the functional profile of cancer-specific T-cells (42, 43); it is also likely to play a pivotal role in whether an antigen breaks self-tolerance and stimulates a robust immune response. Several approaches have attempted to improve natural HLA class I-presented peptides and enhance their immunogenicity. These methodologies revolve around either improving peptide interactions with the HLA or the TCR.

### Improving Peptide Antigens by Enhancing MHC Binding

One relatively easy way to improve the antigenicity of a peptide is to substitute the residues at primary HLA anchors if these residues are suboptimal. The introduction of substitutions at HLA anchor residue positions used to be a popular approach for developing TAA-derived peptides with enhanced immunogenicity for vaccination. This approach is relatively simple as peptide binding motifs for the common HLAs have been established for over 20 years. Most work has been undertaken with HLA A2-restricted peptides where it is known that the majority of tumour-derived peptides do not contain an ideal HLA binding consensus. The use of anchor-modified “heteroclitic” peptides in these systems is widespread [reviewed in Cole et al. (44)]. The best-known example of such a heteroclitic peptide is derived from the Melan-A/MART-1 where the natural 10-mer epitope is EAAGIGILTV, as used in this study. This natural sequence does not have an optimal anchor residue at p2 for the HLA A2 restriction element. Substitution of the p2 Alanine residue for the optimal Leucine residue (45) produces the peptide E**L**AGIGILTV which was shown to have considerably higher immunogenicity *in vitro* (30) and to activate melanoma-reactive CD8 T-cells following vaccination (46). While vaccination with the E**L**AGIGILTV peptide was shown to induce much larger populations of antigen-specific T-cells, these cells were inferior to the T-cells induced by the natural sequence (47). T-cells induced by vaccination with EAAGIGILTV and E**L**AGIGILTV are clonotypically distinct (48). This difference was thought to be the cause of the poor clinical results with the E**L**AGIGILTV analogue (35). Subsequent work in our laboratory demonstrated that TCRs can differentiate between natural and E**L**AGIGILTV anchor-modified peptides enabling T-cells to exhibit a strong preference for either type of antigen (44). Structural studies have confirmed that TCRs can distinguish between EAAGIGILTV and E**L**AGIGILTV peptides (15, 49). This cautionary tale demonstrates that T-cell clonotypes induced by any APL-based immune intervention should be carefully evaluated after *ex vivo* priming to ensure efficacy prior to studies *in vivo*.

### Improving Antigens by Enhancing TCR Binding

An alternative approach for improvement of T-cell epitopes from TAA is to enhance their engagement with cognate TCRs. Alteration of TCR contact residues has the advantage that the resultant APL are highly likely to be viewed as “nonself” and may therefore have increased potential for breaking immune tolerance. However, as for the above-mentioned peptide anchor residue modification, the use of an APL also runs the risk of inducing clonotypes that are ineffective at recognising the native sequence. PS-CPL have been explored as a way of selecting such “mimitopes” (19, 33, 50). This approach relies on the fact that individual T-cells are capable of recognising millions of different peptides (50–52). Consequently, the chance of the natural antigen being optimal for any given TCR is extremely small. PS-CPL data can be combined with computational approaches to predict the range of agonists that are recognised by any given T-cell (50, 53). We recently used this technique to generate a non-natural D-amino acid peptide that could be used to protect humanised mice against a lethal challenge with influenza virus (54). The best D-peptide agonist bore little resemblance to the optimal L-peptide, underscoring the potential scope of TCRs to recognise a wide range of chemically different ligands. These results also highlight that it is currently impossible to predict TCR ligands without undertaking functional screening experiments. PS-CPL approaches have been applied to cancer vaccination (19). A cutaneous T-cell lymphoma (CTCL)-reactive HLA B8-restricted CD8 T-cell clone was used to generate a mimitope that induced populations of T-cells that lysed tumour cells *in vitro* (19). Vaccination of two HLA B8^+^ CTCL patients with this mimitope induced initial tumour regression in both patients (55) but direct comparisons with the natural sequence, as undertaken in this study, were not possible as the targeted TAA was unknown. Substitution of the HLA A2-restricted, human carcinoembryonic antigen (CEA)-derived epitope YLSGANLNL at position 6 (believed to be a TCR contact residue), to produce YLSGA**D**LNL, increased immunogenicity by two orders of magnitude when tested *in vivo* (56). Unfortunately, it was subsequently shown that most T-cells induced by the YLSGA**D**LNL peptide recognised the natural antigen poorly (57). Previous attempts to improve TAA peptides have treated T-cells at the population level rather than focussing on the most effective clonotype(s) within the population. A more promising strategy to improve antigens for cancer vaccination might require identification of the most effective clonotypes and trying to specifically induce such T-cells. This approach, christened ‘TCR Optimised Peptide Skewing Of the Repertoire of T-cells’ (TOPSORT) was used to generate a Melan-A/MART-1 analogue peptide, **F**A**T**GIGI**I**TV that primed larger populations of T-cells then primed with the E**L**AGIGILTV analogue in parallel in 6 of 10 donors as described in the Results section (31). While these results were promising, the approach only improved responses using a minority of HLA A2^+^ PBMC. This deficiency was put down to there being a lack of the required clonotype(s) within the PBMC where the approach failed to show improvements (31). For the TOPSORT approach to be considered for clinical use, it would require an APL that was capable of generating improved T-cell responses in the majority–and preferably all–individuals. Here, we sought to improve this approach by starting with clonotypes that were derived from TIL that were used to induce a complete lasting remission in a metastatic melanoma patient.

### A Super-Agonist for a T-Cell Clone From a TIL Success Primed a Larger EAAGIGILTV-Specific T-Cell Response Than the Natural Sequence From all 14 Healthy Donors Tested

As described above our previous attempt to skew the repertoire of T-cells toward those more effective at recognising melanoma cells used the MEL5 T-cell clone that was grown from a healthy donor to design an APL for T-cell priming was only partially successful. This failure was attributed to some donors lacking the relevant clonotypes that could be stimulated by the APL and cross-react with the natural epitope presented at the melanoma cell surface. Here, we refined the TOPSORT approach by selecting a T-cell clone derived from the TIL infusion product of a melanoma patient that was cured by TIL therapy and that persisted in the patient blood long after complete remission. A PS-CPL screen of the ST8.24 T-cell clone was used to generate a ranked list of predicted super-agonist peptides. The top 10 predicted peptides were tested for their ability to prime an HLA A2-EAAGIGILTV tetramer positive T-cell population from the blood of five healthy donors. An APL of sequence **MTSA**IGIL**P**V was observed to induce a significantly larger T-cell population than parallel assay with the natural EAAGIGILTV in 5/5 donors (Figure 3). We next examined how this peptide expanded EAAGIGILTV-specific T-cells over 14 and 28 days from seven more HLA A2^+^ individuals and found that the **MTSA**IGIL**P**V APL induced substantially more HLA A2-EAAGIGILTV tetramer positive cells. CSFE proliferation assays showed that EAAGIGILTV-specific T-cells stimulated with **MTSA**IGIL**P**V peptide exhibited enhanced proliferation compared to those primed with the natural antigen in parallel (Figure 4). We were unable to look at longer timepoints than 28 days as some cells started to die after 30 days, most likely due to a requirement for restimulation through the TCR.

### MTSAIGILPV Super-Agonist Peptide Exhibits Improved Binding to HLA A2

The **MTSA**IGIL**P**V peptide was shown to exhibit superior binding to HLA A2 compared to the natural EAAGIGILTV sequence. Structural analysis attributed this enhanced binding to the longer side chain of threonine at peptide residue 2 (compared to alanine in EAAGIGILTV) being able to extend deeper into the HLA A2 B-pocket. This increased interaction with the restricting HLA is likely to contribute to why the **MTSA**IGIL**P**V exhibits increased immunogenicity in all 14 donors and both patients tested. Despite the very different sequence **MTSA**IGIL**P**V was shown to be a close structural mimic of EAAGIGILTV, providing an explanation for the strong cross-reactivity between these ligands. Indeed, both peptides induced EAAGIGILTV-specific TCRs that predominantly used the TRAV12-2 gene (Supplementary Figure 6), likely due to the high level of similarity in antigenic features in both peptides, revealed from the crystal structures. Structural studies have demonstrated that the predominant contacts between a TRAV12-2 EAAGIGILTV-specific TCR were made with germline encoded CDR regions. This mode of binding explains the biased usage of TRAV12-2 by EAAGIGILTV-specific TCRs and may provide an explanation for why T-cells induced with the **MTSA**IGIL**P**V peptide cross-react with the EAAGIGILTV peptide. The increased HLA binding of the **MTSA**IGIL**P**V peptide is unlikely to explain why the T-cells primed from the PBMC of a melanoma patient prior to treatment with this APL were better at recognising the autologous melanoma line than those induced by the natural antigen.

### EAAGIGILTV Specific T-Cells Bind HLA A2-MTSAIGILPV With Increased Avidity

In addition to the improved binding of the **MTSA**IGIL**P**V peptide to HLA A2, clonal, and polyclonal T-cells bound HLA A2-**MTSA**IGIL**P**V tetramer with greater intensity, suggesting that the associated TCRs recognised the agonist peptide with increased affinity compared to HLA A2-EAAGIGILTV. Taken together, these data suggest that T-cells received greater antigenic stimulus upon priming with **MTSA**IGIL**P**V peptide. This enhanced stimulus presumably accounts for the increased T-cell expansion observed across all 16 subjects used for this study compared to the natural EAAGIGILTV sequence in parallel assays. Further structural and biophysical studies will be required to formally confirm that the **MTSA**IGIL**P**V peptide binds more readily to cognate TCRs.

### MTSAIGILPV Primed T-Cells From Blood That Exhibit Improved Killing of Melanoma Tumour

Importantly, the greater population of EAAGIGILTV-specific T-cells expanded from the PBMCs of healthy donors and a melanoma patient prior to treatment, using the **MTSA**IGIL**P**V peptide, exhibited superior killing of cancer cells. Thus, this APL was able to induce T-cells in greater *quantity*. In one case we were able to demonstrate that the **MTSA**IGIL**P**V induced T-cells of greater *quality* on a per cell basis.

### Summary

In summary, we used a T-cell clone derived from the TIL of a melanoma patient that persisted in patient PBMC after long-lasting tumour remission to generate a novel APL super-agonist peptide with the sequence **MTSA**IGIL**P**V. This APL was capable of inducing greater numbers of T-cells specific for the natural antigen EAAGIGILTV from the PBMC of 14/14 healthy donors and 2/2 patients tested compared to the natural antigen itself. Importantly, the APL-generated T-cells were shown to kill tumour cells with greater potency than those induced by the natural epitope. The greater proliferation of cognate T-cells induced by the **MTSA**IGIL**P**V sequence might reflect its greater binding to HLA. This peptide also engages host TCRs with better efficiency and thereby could encompass an enhanced ability to break self-tolerance mechanisms. The differences in EAAGIGILTV-specific clonotypes induced by **MTSA**IGIL**P**V suggests that this APL is viewed differently by TCRs despite its structural similarity. Further work will be required to determine how this sequence has potential to induce T-cells in greater quantity that can exhibit enhanced function at the single cell level. Overall, our data suggest that the **MTSA**IGIL**P**V sequence might make a promising candidate for peptide vaccination of HLA A2^+^ melanoma patients.

## Ethics Statement

This study was carried out in accordance with the recommendations of the Scientific Ethics Committee for the Capital Region of Denmark. All subjects gave written informed consent in accordance with the Declaration of Helsinki. The protocol was approved by the Scientific Ethics Committee for the Capital Region of Denmark.

## Author Contributions

SG, GD, MA, AW, AF, CR, VB, ST, AL, MC, IS, MD, DC, BS, PR, and AS performed and/or directed experiments, analysed data, and critiqued the manuscript. IS and MD provided patient samples. GD and AS conceived, funded, and directed the project. SG, GD, and AS wrote the manuscript.

### Conflict of Interest Statement

DC and AL were employed by Immunocore Limited. The altered peptide ligands disclosed in this manuscript are the subject of a pending patent application from Cardiff University. The remaining authors declare that the research was conducted in the absence of any commercial or financial relationships that could be construed as a potefgene-10-00127ntial conflict of interest.
