# Supplementary material for: Peptide Super-Agonist Enhances T-Cell Responses to Melanoma
Source: Front Immunol. 2019 Mar 13;10:319. doi: 10.3389/fimmu.2019.00319 (PMC6425991; doi:10.3389/fimmu.2019.00319)
Supplement: Supplementary file 1 [file Data_Sheet_1.pdf]

Supplementary Material

Peptide Super-agonist enhances T-cell Responses to Melanoma

Sarah A. E, Galloway†, Garry Dolton†, Meriem Attaf, Aaron Wall, Anna Fuller, Cristina Rius, Valentina Bianchi, Sarah Theaker, Angharad Lloyd, Marine E. Caillaud, Inge Marie Svane, Marco Donia, David K. Cole, Barbara Szomolay, Pierre Rizkallah and Andrew K. Sewell*

† These authors contributed equally

*** Correspondence:** Andrew Sewell: sewellak@cardiff.ac.uk

Supplementary Figures 1-4

Supplementary Figure 1: Associated flow cytometry plots from Melan-A tetramer staining of $CD8^+$ T-cells primed with candidate super-agonist peptides. PBMCs were extracted from healthy buffy coat obtained from the Welsh Blood Service, using a SepMate protocol. CD8 positive T-cells were extracted and co-incubated, at a density of 3 $x10^6$ per mL, with 7 $x10^6$ irradiated autologous CD8 negative cells pulsed for 1 hour with indicated peptide for two weeks. The number of $CD8^+$, HLA A2-EAAGIGILTV $tetramer^+$ cells from each condition is expressed as a percentage.

Supplementary Figure 2: Associated flow cytometry plots of EAAGIGILTV-tetramer stained CD8 T-cells primed with super-agonist peptide **MTSA**IGIL**P**V and wild type peptide EAAGIGILTV for 14 days. CD8 T-cells from 7 healthy HLA $A2^+$ donors were isolated from healthy PBMCs and cultured for 14 days alongside autologous CD8 negative cells primed with EAAGIGILTV or **MTSA**IGIL**P**V peptides. The number of $CD8^+$, HLA A2-EAAGIGILTV $tetramer^+$ cells from each condition is expressed as a percentage.

Supplementary Figure 3: As for Supplementary Figure 2 but after 28 days.

Supplementary Figure 4: (A) Priming of purified CD8 T-cells from healthy donor number 13 with wild-type EAAGIGILTV (Melan-A) and super-agonist **MTSA**IGIL**P**V peptides, followed by tetramer staining with EAAGIGILTV and ALWGPDPAAA (irrelevant, from preproinsulin) epitopes. An optimal tetramer staining protocol was used that included a PE conjugated secondary antibody according to the material and methods. **(B)** Phenotypic analyses of EAAGIGILTV and **MTSA**IGIL**P**V primed CD8 T-cells from the same donor. Gated on EAAGIGILTV $tetramer^+$ cells followed by display for the various phenotypic markers. Fluorescence minus one controls were used to set the gates. CD8 T-cells from freshly isolated PBMCs were stained in parallel to aid analysis and shown in grey (contour plot with outliers shown). The EAAGIGILTV $tetramer^+$ cells are overlaid using large dots for display, with the percentage of cells residing in each quadrant shown. There was no major difference between the EAAGIGILTV or **MTSA**IGIL**P**V lines based on the markers used.

Supplementary Figure 5: Supporting flow cytometry data of HLA A2 staining from T2 cell binding assays shown in Figure 6A. 5 x10^4 T2 cells (TAP deficient HLA $A2^+$) were cultured in AIM-V serum-free media with indicated peptides at concentrations of 1 µM, 10 µM and 100 µM. Assays performed in duplicate. The following day, cells were stained with FITC-conjugated HLA A2 antibody and LIVE/DEAD vivid (to allow exclusion of dead cells). Results are expressed as the MFI of HLA A2 expression, with the dashed line indicating base line HLA A2 expression of T2 cells untreated with peptide.

Supplementary Figure 6: Priming CD8 T-cells with MTSAIGIL**P**V **elicits different CD8 T-cell clonotypes and only 3/162 shared CDR3 regions.** (**A**) CDR3 analysis of alpha (left) and beta TCR chains (right) from donor 9 CD8 T-cells single cell sorted on EAAGIGILTV-tetramer positivity, where each segment of the pie represents a distinct clonotype. Shared CDR3 sequences between the two priming conditions (**MTSA**IGIL**P**V or EAAGIGILTV peptide) are highlighted. (**B**) TCR variable (V) and joining (J) gene pairing analysis of alpha (left) and beta TCR genes (right), from donor 9 CD8 T-cells primed with **MTSA**IGIL**P**V or EAAGIGILTV and single cell sorted on EAAGIGILTV tetramer positivity. Dominant gene pairings highlighted in bold.

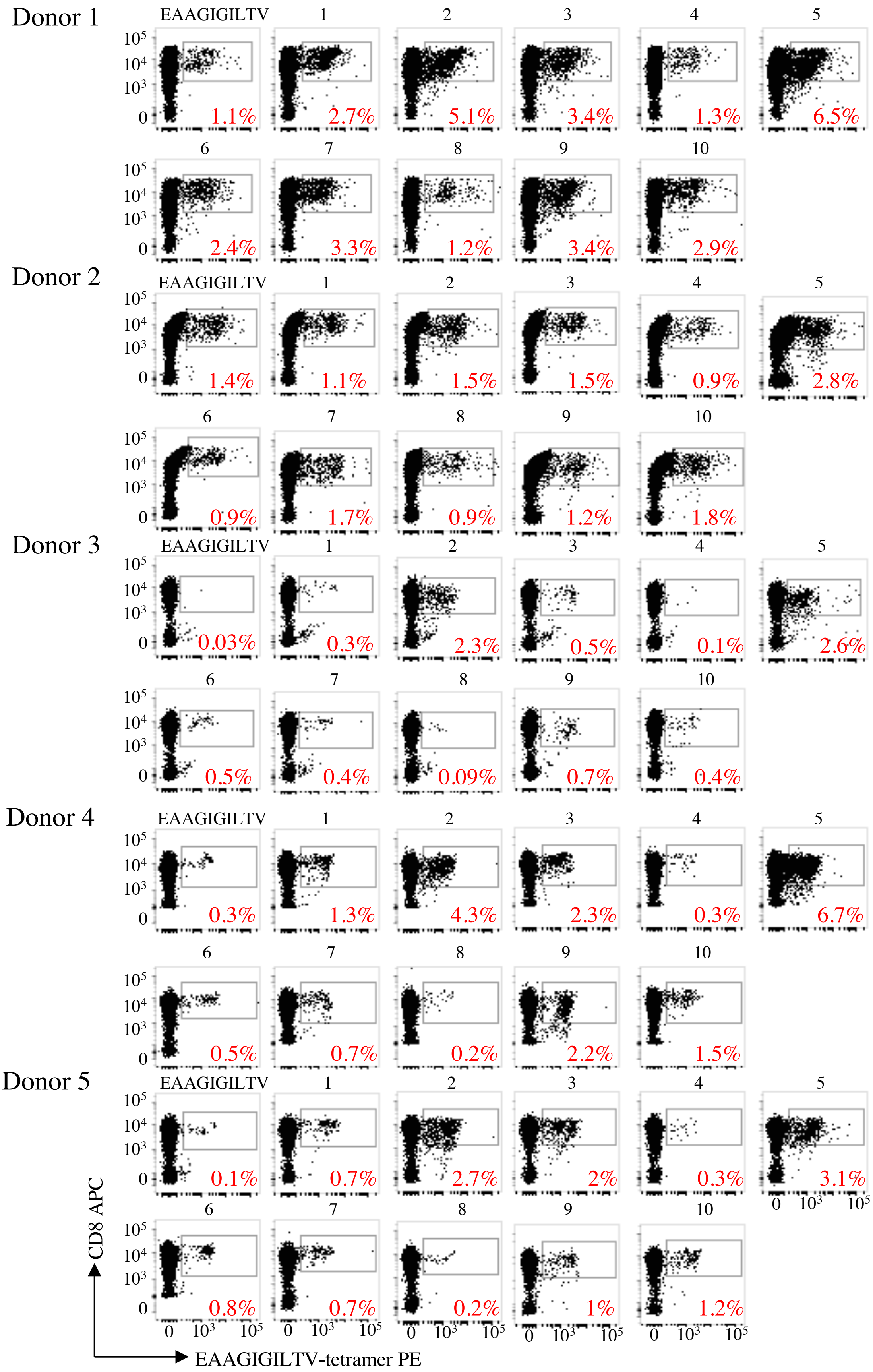

Supplementary Figure 1: Associated flow cytometry plots from Melan-A tetramer staining of CD8+ T-cells primed with candidate super-agonist peptides. PBMCs were extracted from healthy buffy coat obtained from the Welsh Blood Service, using a SepMate protocol. CD8 positive T-cells were extracted and co-incubated, at a density of 3 x10^6 per mL, with 7 x10^6 irradiated autologous CD8 negative cells pulsed for 1 hour with indicated peptide for two weeks. The number of CD8+, HLA A2-EAAGIGILTV tetramer+ cells from each condition is expressed as a percentage.

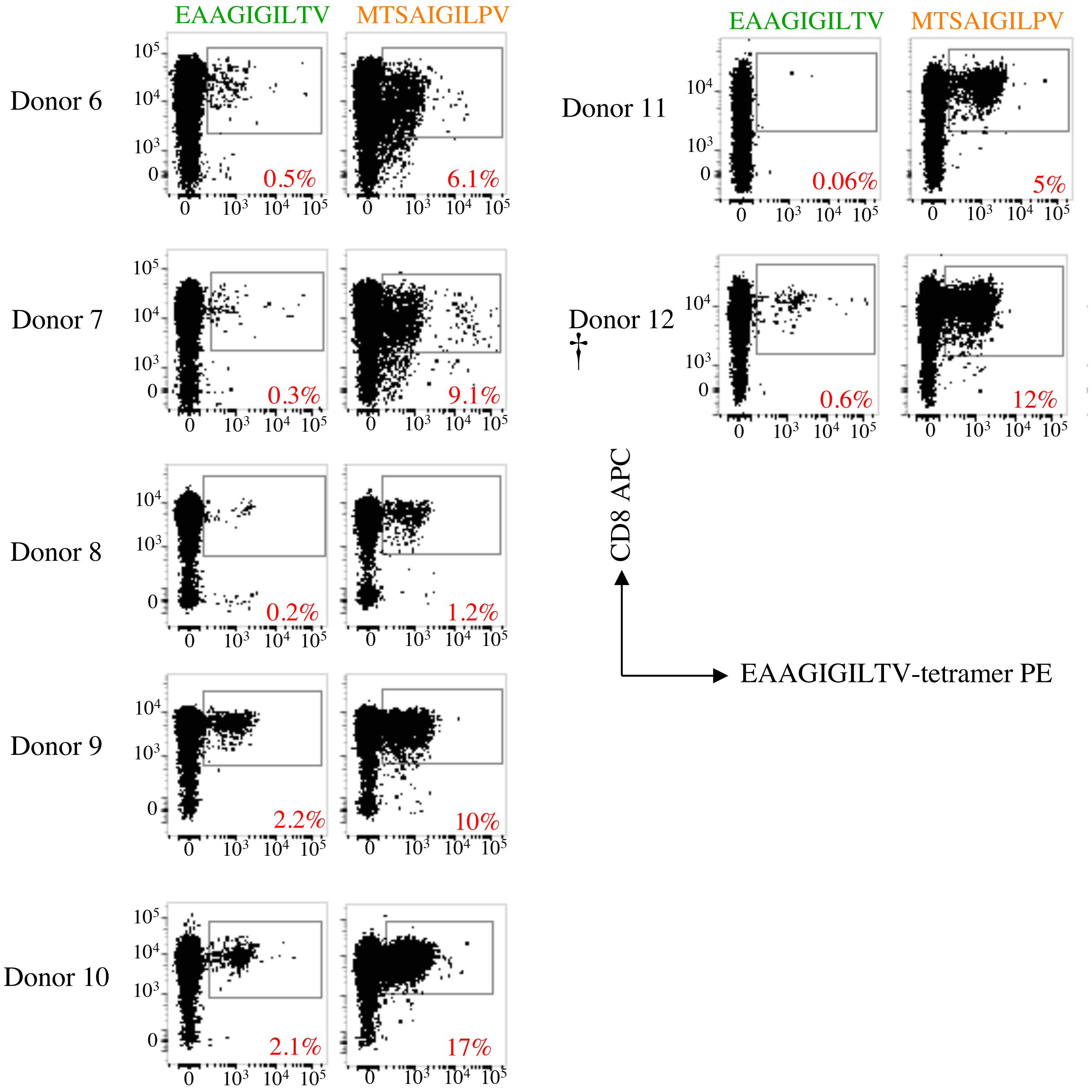

Supplementary Figure 2: Associated flow cytometry plots of EAAGIGILTV-tetramer stained CD8 T-cells primed with super-agonist peptide **MTSA**IGIL**P**V and wild type peptide EAAGIGILTV for 14 days. CD8 T-cells from 7 healthy HLA A2^{+} donors were isolated from healthy PBMCs and cultured for 14 days alongside autologous CD8 negative cells primed with EAAGIGILTV or **MTSA**IGIL**P**V peptides. The number of CD8^{+}, HLA A2-EAAGIGILTV tetramer^{+} cells from each condition is expressed as a percentage.

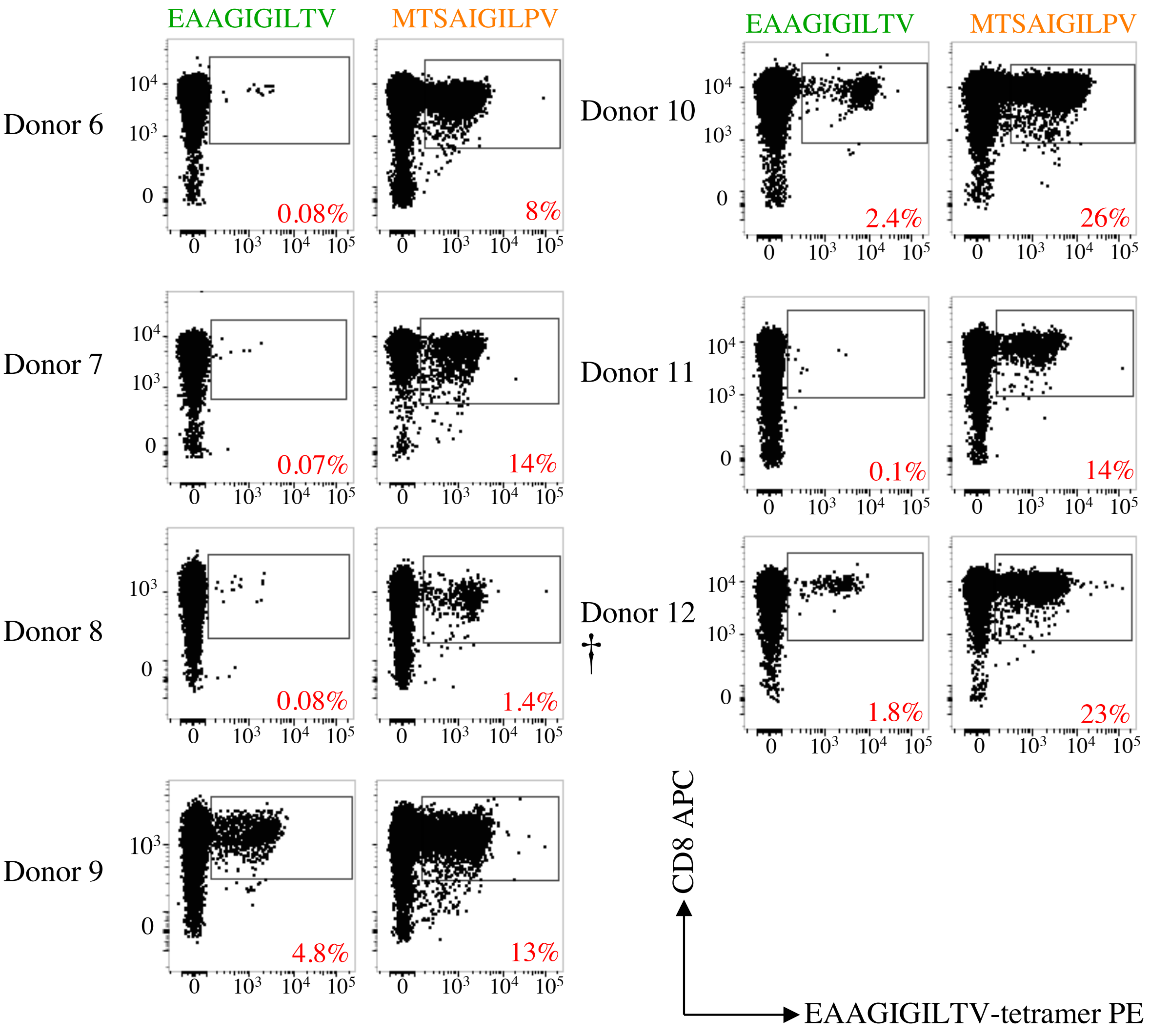

Supplementary Figure 3: As for Supplementary Figure 2 but after 28 days.

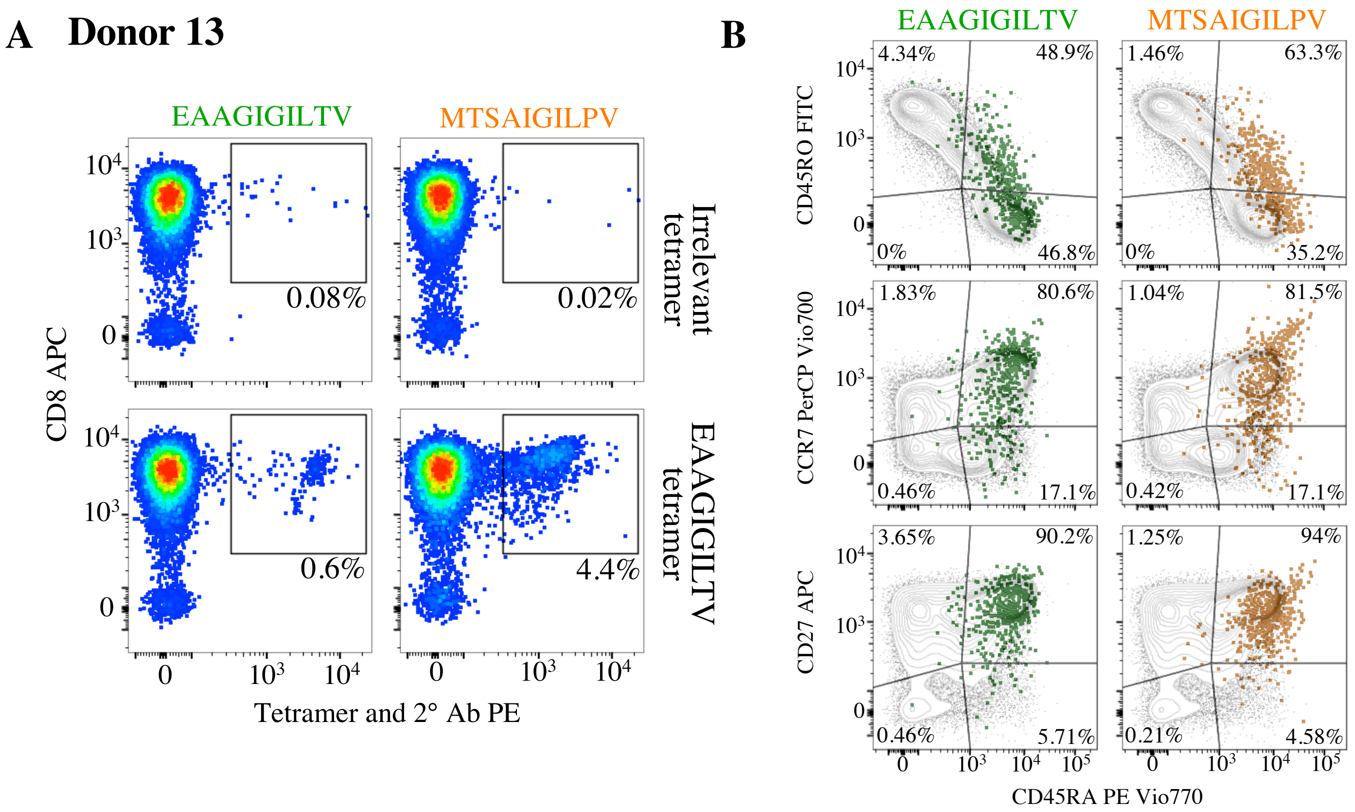

Supplementary Figure 4: (A) Priming of purified CD8 T-cells from healthy donor number 13 with wild-type EAAGIGILTV (Melan-A) and super-agonist **<u>MTSA</u>**IGIL**<u>P</u>**V peptides, followed by tetramer staining with EAAGIGILTV and ALWGPDPAAA (irrelevant, from preproinsulin) epitopes. An optimal tetramer staining protocol was used that included a PE conjugated secondary antibody according to the material and methods. **(B)** Phenotypic analyses of EAAGIGILTV and **<u>MTSA</u>**IGIL**<u>P</u>**V primed CD8 T-cells from the same donor. Gated on EAAGIGILTV tetramer^{+} cells followed by display for the various phenotypic markers. Fluorescence minus one controls were used to set the gates. CD8 T-cells from freshly isolated PBMCs were stained in parallel to aid analysis and shown in grey (contour plot with outliers shown). The EAAGIGILTV tetramer^{+} cells are overlaid using large dots for display, with the percentage of cells residing in each quadrant shown. There was no major difference between the EAAGIGILTV or **<u>MTSA</u>**IGIL**<u>P</u>**V lines based on the markers used.

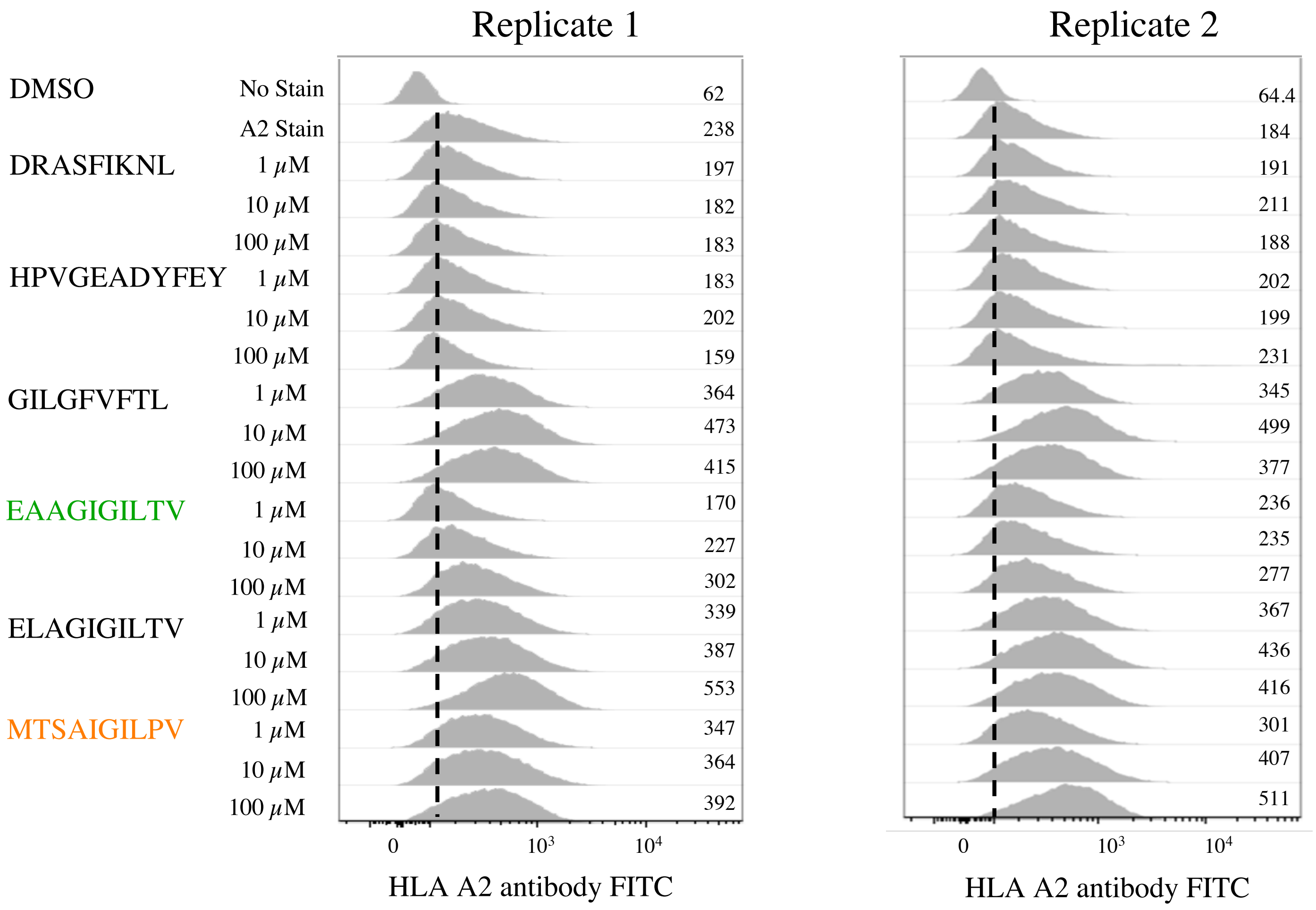

Supplementary Figure 5: Supporting flow cytometry data of HLA A2 staining from T2 cell binding assays shown in Figure 6A. 5 x10^4 T2 cells (TAP deficient HLA A2$^+$) were cultured in AIM-V serum-free media with indicated peptides at concentrations of 1 μM, 10 μM and 100 μM. Assays performed in duplicate. The following day, cells were stained with FITC-conjugated HLA A2 antibody and LIVE/DEAD vivid (to allow exclusion of dead cells). Results are expressed as the MFI of HLA A2 expression, with the dashed line indicating base line HLA A2 expression of T2 cells untreated with peptide.

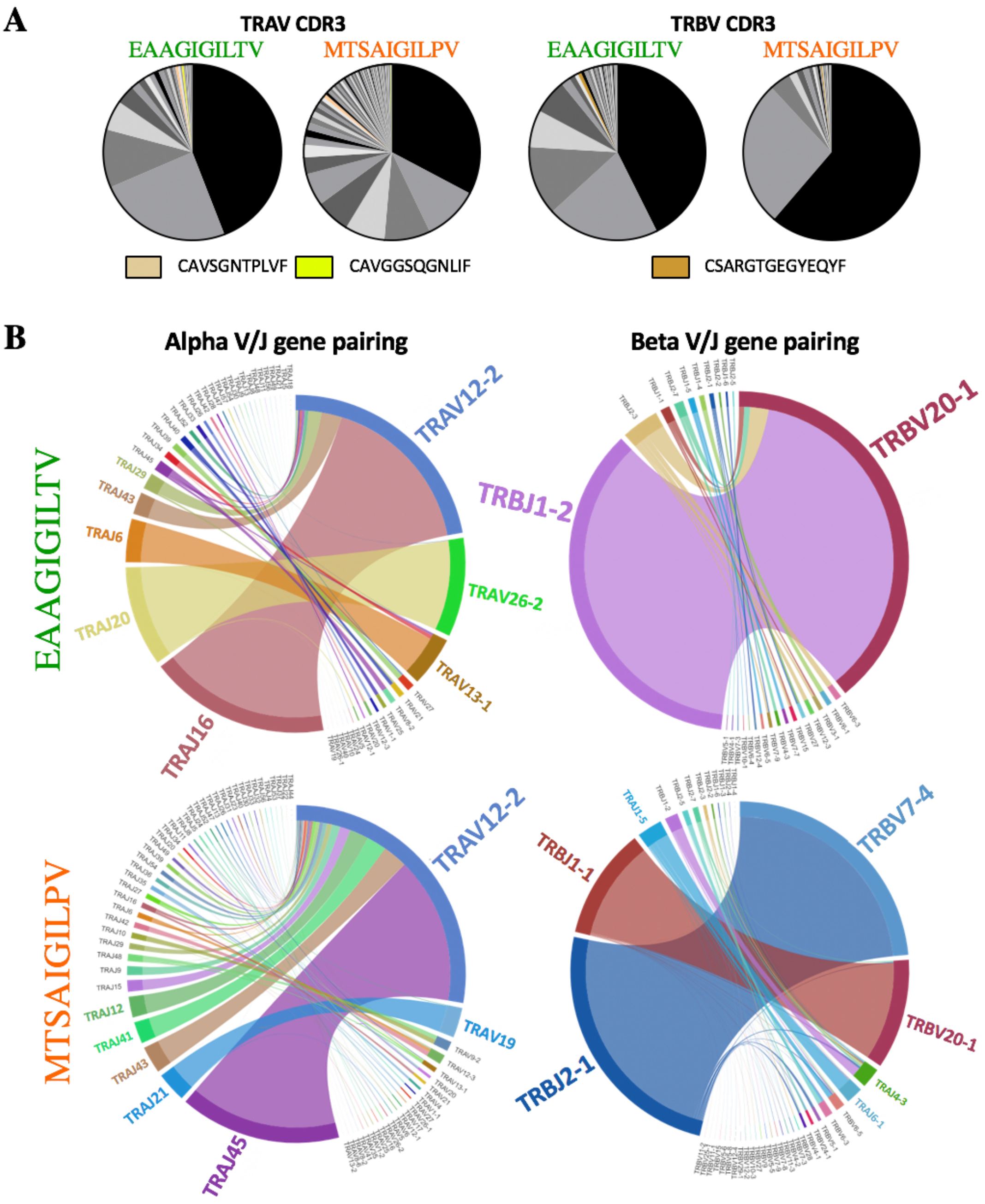

Supplementary Figure 6: Priming CD8 T-cells with MTSAIGILPV elicits different CD8 T-cell clonotypes and only 3/162 shared CDR3 regions. (**A**) CDR3 analysis of alpha (left) and beta TCR chains (right) from donor 9 CD8 T-cells single cell sorted on EAAGIGILTV-tetramer positivity, where each segment of the pie represents a distinct clonotype. Shared CDR3 sequences between the two priming conditions (**MTSA**IGIL**P**V or EAAGIGILTV peptide) are highlighted. (**B**) TCR variable (V) and joining (J) gene pairing analysis of alpha (left) and beta TCR genes (right), from donor 9 CD8 T-cells primed with **MTSA**IGIL**P**V or EAAGIGILTV and single cell sorted on EAAGIGILTV tetramer positivity. Dominant gene pairings highlighted in bold.
